# Mapping the Spatial Heterogeneity and Driving Mechanisms of Species-Specific Mangrove Carbon Stock with GF-1 Imagery and Models

**DOI:** 10.3390/s26185787

**Published:** 2026-09-11

**Authors:** Xin Li, Yuxing Wang, Xunan Liu, Lei Zhang, Xiaoyong Shi

**Affiliations:** 1 Key Laboratory of Marine Chemistry Theory and Technology, Ministry of Education, Ocean University of China, Qingdao 266100, China; lixin19950210@163.com; 2 National Marine Hazard Mitigation Service, Beijing 100194, China; yuxing.wang@foxmail.com (Y.W.); liuhui@tst-qd.com (X.L.); zhanglei.ers@gmail.com (L.Z.)

**Keywords:** mangrove carbon stock, biomass model, species classification, allometric relationship

## Abstract

Accurate estimation of mangrove carbon stocks and the driving mechanisms behind their spatial patterns are crucial for blue carbon assessment and management. This study addresses several challenges in remote sensing estimation of mangroves, including the limited generalizability of single estimation models, the difficulty in directly inverting belowground biomass (BGB), and insufficient analysis of the driving mechanisms underlying spatial heterogeneity. Using typical coastal mangrove distribution regions as the study area, an analytical framework integrating species-specific modeling, high-accuracy species classification, and multi-dimensional mechanism analysis was constructed by combining GF-1 remote sensing images with field survey data. First, species-specific aboveground biomass (AGB) and belowground biomass estimation models were established. Subsequently, high-precision mangrove species classification was achieved, with an overall species classification accuracy of 98.18% and a Kappa coefficient of 0.94. Furthermore, a variety of methods—including SHAP analysis, geographical detectors, and structural equation modeling (SEM)—were comprehensively applied to systematically examine the driving mechanisms behind the spatial distribution of carbon stocks. The results indicate that spatial heterogeneity in carbon stocks is primarily driven by spatial characteristics (latitude (Lat) and longitude (Lon), explanatory power = 0.514), with topographic characteristics (DEM, slope, aspect, explanatory power = 0.239) playing a synergistic and enhancing auxiliary role, and significant interactions existing among these factors. This study significantly improves the accuracy of mangrove carbon stock estimation and deepens the understanding of its driving mechanisms, providing scientific methodology and a case study support for refined monitoring, conservation, and carbon sink management of coastal blue carbon ecosystems.

## 1. Introduction

Climate change, characterized primarily by global warming, has become a severe challenge facing human society today [1]. With global carbon emissions continuously rising, the atmospheric CO_2_ concentration reached 423 ppm in 2024, pushing human-induced warming to 1.36 °C and approaching the critical threshold of 1.5 °C [2]. Recent scientific research has revealed dangerous feedback mechanisms within the carbon cycle, as long-term cumulative greenhouse gases persistently weaken the buffering capacity of Earth’s natural carbon sinks [3]. Reports from the International Science Council further indicate that terrestrial carbon sinks are nearing a critical limit, with their absorption capacity declining continuously due to climate change. The ocean, another key carbon sink, is also experiencing reduced efficiency in absorbing CO_2_ [4]. A report indicates that over the past decade, climate change has reduced the efficiency of global land and ocean carbon sinks by 23% and 6%, respectively [5]. Against this backdrop, identifying, protecting, and enhancing natural carbon sink systems that remain highly efficient and stable has become more urgent than ever for mitigating climate change and achieving global carbon neutrality goals.

Among various ecosystems, mangroves—distributed in tropical and subtropical coastal intertidal zones—stand out for their exceptional blue carbon functionality, serving as a natural frontline in combating climate change. Although mangroves cover less than 1% of the world’s forest area, their carbon sequestration capacity per unit area far surpasses that of most terrestrial forests. Research indicates that mangroves are among the most carbon-dense ecosystems globally [6]. Their carbon pools exist in aboveground and belowground vegetation as well as in deep soils, enabling carbon stock for centuries to millennia [7]. Therefore, accurately quantifying mangrove AGB and elucidating the driving mechanisms behind their spatial distribution are not only fundamental for scientifically assessing their climate regulation service value but also prerequisites for promoting nature-based solutions and advancing blue carbon trading to realize the value of ecological products.

Leveraging its strengths in large-scale and periodic observation, remote sensing technology has become a core tool for monitoring mangrove biomass and carbon stock. Some studies have developed various estimation models for mangrove AGB based on multispectral, hyperspectral, and synthetic aperture radar data [8,9]. However, existing studies still face several major challenges. Firstly, most models treat mangroves as a homogeneous entity, adopting a generalized modeling approach that overlooks the significant differences among mangrove species in canopy structure, spectral characteristics, and biomass allocation strategies. This inevitably introduces model errors, particularly in regions with complex species compositions. Secondly, as remote sensing has difficulty directly detecting belowground vegetation structures, there is substantial uncertainty in assessing the overall vegetation carbon pool of mangroves. Additionally, although studies generally recognize significant spatial heterogeneity in mangrove carbon stock, the analysis of its driving mechanisms is often simplistic—either focusing only on single environmental factors or lacking in-depth investigation into the interactions among drivers at different scales. Consequently, it is challenging to reveal the complex hierarchical driving structure underlying these patterns.

To address the above challenges, this study focuses on typical mangrove distribution areas, including the eastern and northern parts of Tieshan Harbor, Shankou Mangrove National Nature Reserve (Shankou MNNR), and Zhanjiang Mangrove National Nature Reserve (Zhanjiang MNNR). By utilizing high spatial resolution satellite remote sensing with rich spectral and texture features, combined with field measured data and multi-model fusion strategies, a species adaptive analysis framework was constructed. The specific objectives are as follows: (1) to establish species-specific AGB remote sensing estimation models and derive BGB estimation models based on allometric relationships; (2) to achieve high-precision classification of mangrove species, enabling a comprehensive assessment of the total vegetation carbon pool; and (3) to systematically analyze the driving mechanisms of spatial patterns in mangrove carbon stock.

We hypothesize that species-specific biomass modeling can reduce estimation errors. Spatial factors dominated by Lat and Lon and topographic factors dominated by DEM, slope and aspect affect the spatial variation of carbon stock in mangroves. Through this systematic research, we aim not only to provide a high-spatial-resolution map of mangrove carbon stock distribution for the study area but also to offer new methodological insights and case support for improving remote sensing estimation models of mangrove biomass and deepening the understanding of spatial heterogeneity patterns in coastal ecosystems.

## 2. Materials and Methods

### 2.1. Research Area

**Figure 1:**
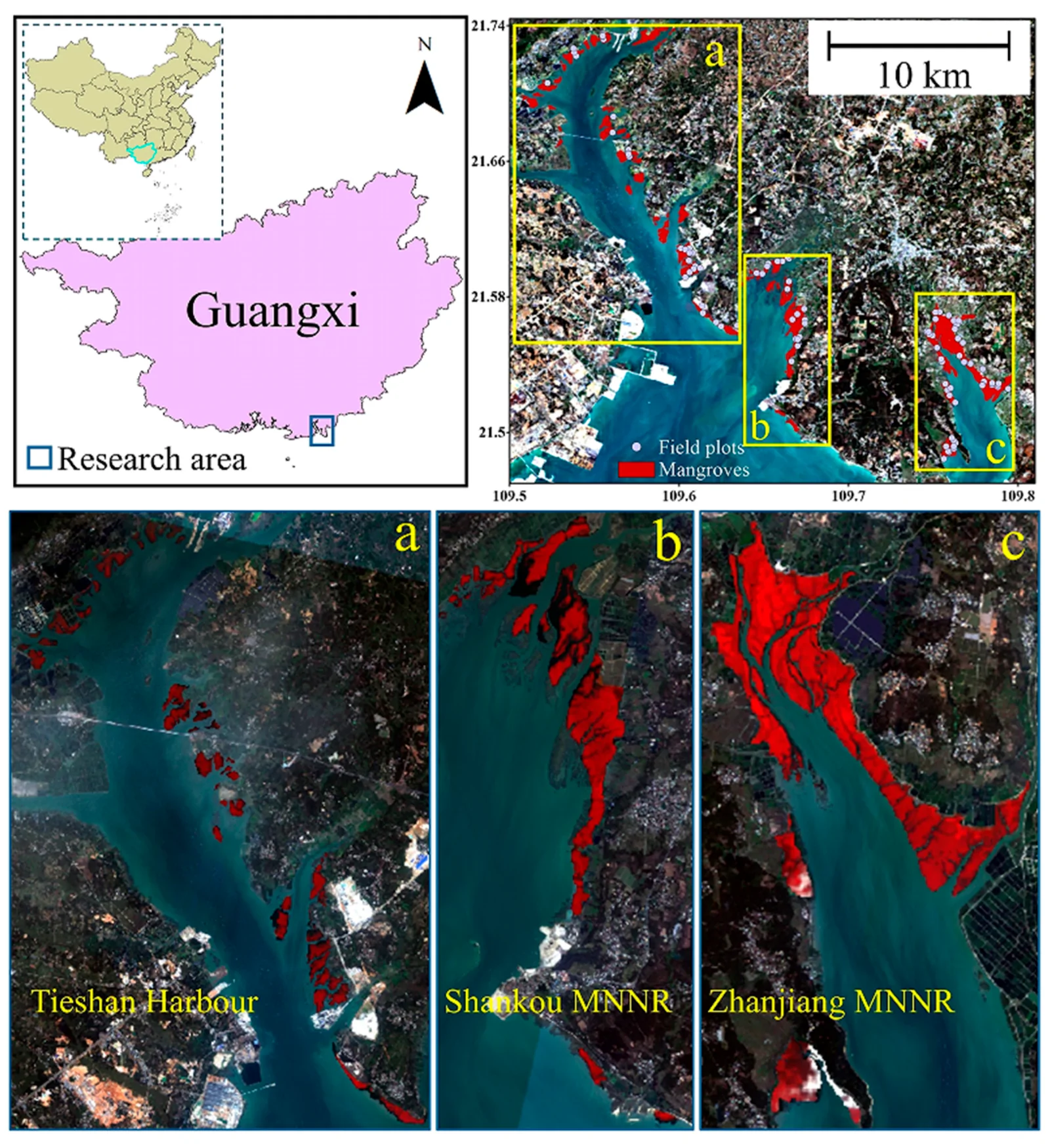
Location of the research area and mangrove distribution. Note: (**a**) Tieshan Harbor; (**b**) Shankou MNNR; (**c**) Zhanjiang MNNR.

The geographical location of the study area is shown in Figure 1. The study area covers the eastern and northern parts of Tieshan Harbor, Shankou MNNR, and Zhanjiang MNNR, with a precise geographical range of 21.45–21.75° N latitude and 109.5–109.8° E longitude. The entire study area features a subtropical monsoon climate, with hot, rainy summers and mild, relatively dry winters. The region receives abundant precipitation, with an annual average rainfall of 1500–1800 mm, and more than 80% of the annual rainfall occurs from April to September during the growing season, providing sufficient water supply for mangrove development [10]. The region is frequently affected by tropical cyclones from summer to early autumn, which substantially contributes to the formation of coastal geomorphology and vegetation structure. This study focuses on the Tieshan Harbor area, which comprises intertidal mudflats and estuaries that provide suitable habitats for mangrove propagation. Shankou MNNR is renowned for its well-preserved and continuous stretches of mangrove forests with high species diversity of true mangrove plants. As the largest mangrove reserve in China, Zhanjiang MNNR features a complex and indented coastline, where some sites are characterized by elevated salinity and intense wave action [11].

The mangrove habitats in the study area are mainly covered by coastal solonchak and mangrove swamp soil. These typical wetland soils possess deep sediment layers, fine silt texture, and high organic carbon content, with weak permeability and long-term anaerobic conditions, which greatly facilitate the carbon sequestration and accumulation of mangrove ecosystems. Based on previous regional literature investigations on coastal wetland environments, the surface soil salinity of local mangrove habitats generally ranges from 12‰ to 28‰ [10]. Affected by tidal flooding and terrestrial freshwater input, soil salinity exhibits obvious spatial heterogeneity, with offshore tidal flat areas maintaining relatively stable and higher salinity, while nearshore and estuarine areas show lower and more variable salinity levels.

### 2.2. Research Data

#### 2.2.1. Field Survey Data Acquisition and Processing

**Figure 2:**
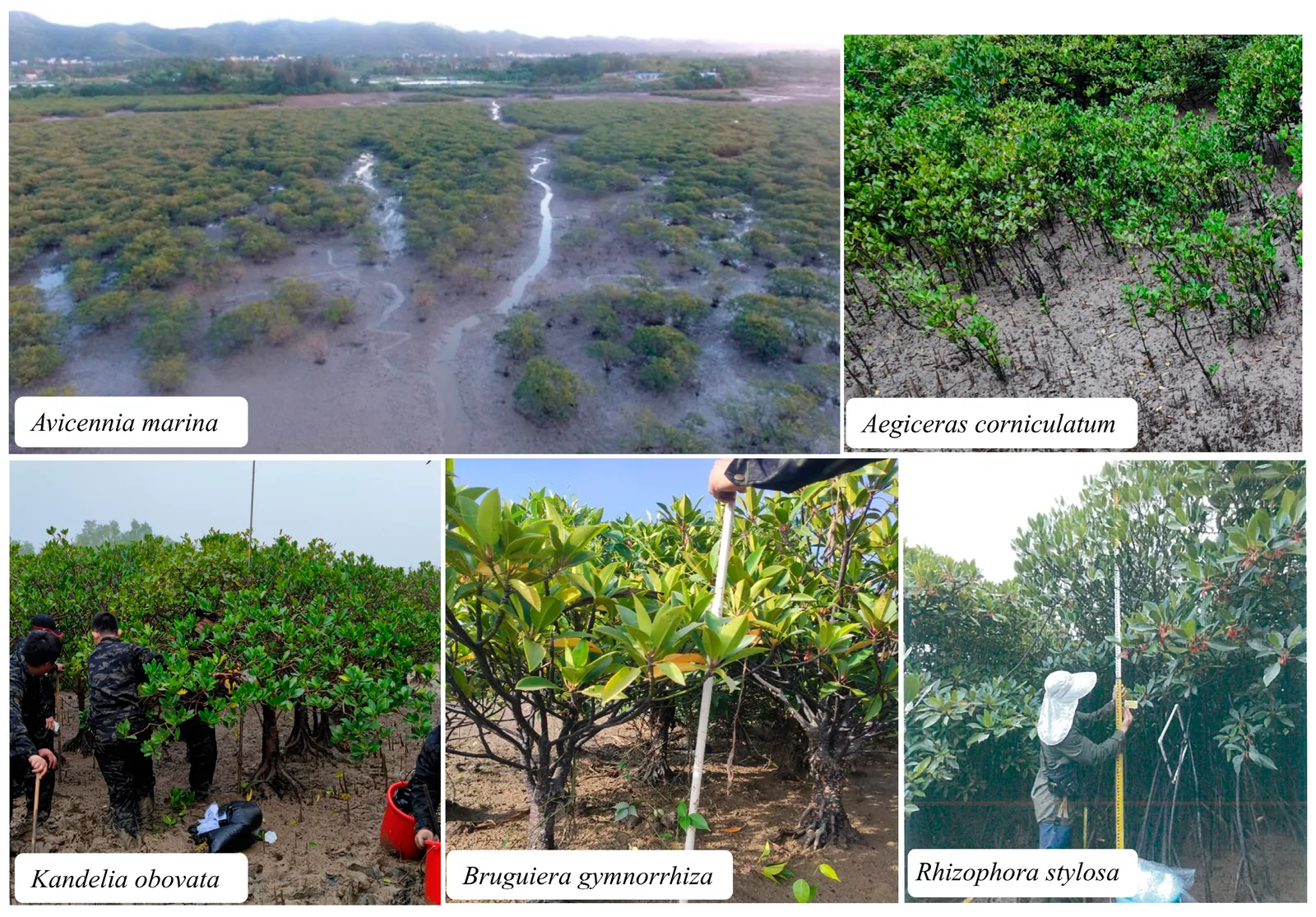
Field survey of mangrove species in the study area.

A field survey was conducted in July 2024 using a purposive sampling method to collect data from 129 quadrats (each 10 × 10 m) (Figure 1), obtaining samples of mangrove biophysical characteristics. We stratified the entire mangrove distribution area based on mangrove species types, tidal elevation gradients and vegetation coverage levels. In each stratified unit, we manually located representative plots to cover the growth density and habitat conditions of all mangroves, while deliberately avoiding mixed-species intermingled zones. This sampling approach ensured that each quadrat contained only a single species or a certain species as the dominant species, thereby minimizing the influence of species variation on subsequent biomass and carbon stock calculations. It should be noted that this purposive sampling strategy, which excludes mixed-species transition zones, is beneficial for developing reliable species-specific allometric models, but it may limit the representativeness for real-world mixed-canopy pixels when these models are extended to all image pixels. Fieldwork included using a Global Navigation Satellite System (GNSS) receiver to obtain plot coordinates, measuring diameter at breast height with a steel diameter tape and tree height with a laser hypsometer, and identifying mangrove species. All trees within each quadrat were systematically measured. Field surveys were conducted during low tide; for trees obscured by dense branches, multiple observation distances and angles were adopted to guarantee accurate height readings. Sample data were ultimately obtained for five mangrove species: *Avicennia marina* (*A. marina*), *Bruguiera gymnorrhiza* (*B. gymnorrhiza*), *Rhizophora stylosa* (*R. stylosa*), *Aegiceras corniculatum* (*A. corniculatum*), and *Kandelia obovata* (*K. obovata*) (Figure 2). Following the Chinese industry standard “Technical Regulations for Investigation and Assessment of Carbon Storage in Mangrove Ecosystems” [12], species-specific allometric equations were used to calculate the AGB and BGB of each tree. The total biomass was calculated as the sum of AGB and BGB. Table 1 summarizes the allometric equations used for the different mangrove species.

#### 2.2.2. Remote Sensing Data Extraction and Processing

This study employed Gaofen-1 (GF-1) satellite remote sensing imagery to extract band indices, vegetation indices, and texture features as independent variables for model construction (Table 2). The GF-1 satellite was launched in 2013 and is equipped with two types of sensors critical for land and vegetation monitoring: a 2-m panchromatic (PAN) camera and an 8-m multispectral (MSS) sensor. The data consist of four spectral bands: blue (450–520 nm), green (520–590 nm), red (630–690 nm), and near-infrared (770–890 nm) [13]. Cloud-free GF-1 imagery matching the July 2024 field survey period was unavailable due to persistent cloud cover, so we selected cloudless imagery acquired on 22 October 2024, for analysis. It should be acknowledged that there is a two-month time mismatch between field sampling and remote sensing data acquisition, which brings uncertainties to species classification and biomass estimation. The two-month time gap is assumed to have limited influence on this study, as local mangroves are evergreen communities without seasonal leaf shedding; their canopy structure and leaf spectral properties remain stable throughout the summer and autumn growing seasons, and vegetation coverage shows no obvious fluctuations between July and October. Future investigations should strive to arrange field surveys synchronously with cloud-free satellite image acquisition to reduce temporal matching errors. The GF-1 imagery used in this study was acquired on 22 October 2024, with cloud cover of less than 10%. Visual interpretation and screen digitization were performed using ENVI 5.3 based on image interpretation elements to delineate mangrove areas. The delineation results were validated using Google Earth (https://earth.google.com/static/single-threaded/versions/20260820_1201_RC00/index.html?#@0,0,0a,22251752.77375655d,35y,0h,0t,0r/data=CgRCAggBQgIIAEoNCP___________wEQAA, accessed on 8 September 2026) imagery and ground truth data.

To enhance image quality, this study systematically preprocessed the original GF-1 imagery using ENVI 5.3 software. First, radiometric calibration and atmospheric correction were applied separately to the multispectral and panchromatic images to convert the raw digital number (DN) values into surface reflectance data, thereby mitigating sensor- and atmosphere-related interference. Next, orthorectification was performed to correct pixel displacement caused by topographic variation and sensor geometric distortion. Subsequently, image fusion techniques were employed to merge the multispectral and panchromatic images, producing a multispectral image with a spatial resolution of 2 m. The fused image allows clear identification of canopy variation details among mangrove species, providing a reliable basis for subsequent high-precision species classification. Finally, the fused imagery underwent mosaicking, edge blending, and clipping to the study area, resulting in a high-quality image dataset that comprehensively covers the research extent.

Mangroves exhibit distinct spectral responses across different wavelength bands. In the red band, chlorophyll strongly absorbs light, resulting in low vegetation reflectance. In contrast, in the near-infrared band, strong scattering of light by vegetation leaves leads to very high reflectance. This significant reflectance difference makes the near-infrared band highly sensitive to green vegetation, serving as a core basis for constructing vegetation indices to estimate biomass. The following vegetation indices have been widely used in estimating mangrove AGB: Normalized Difference Vegetation Index (NDVI), Soil-Adjusted Vegetation Index (SAVI), Enhanced Vegetation Index (EVI), Difference Vegetation Index (DVI), Simple Ratio Vegetation Index (RVI), and Green Normalized Difference Vegetation Index (GNDVI).

Textural features refer to the patterns and characteristics formed by the repetitive appearance of fine-scale objects in an image. Based on the gray-level co-occurrence matrix (GLCM) principle, eight textural features were extracted from the remote sensing imagery: Mean (ME), Variance (VA), Homogeneity (HO), Contrast (CO), Dissimilarity (DI), Entropy (EN), Second Moment (SE), and Correlation (COR). Previous studies have identified certain correlations among the same textural features across different bands. Therefore, principal component analysis (PCA) was employed to reduce the dimensionality of the textural feature values from the four bands, obtaining new textural variables for model construction. It is worth noting that for each texture feature, independent principal component analysis is conducted, and the corresponding four-band original values are compressed into two principal components. For every texture feature, the first two principal components cumulatively explained more than 80% of the total variance, which was our criterion for retaining two components per feature. PCA is a dimensionality reduction method widely applied to large datasets to enhance computational efficiency while retaining the information they carry [20]. After performing PCA on the four bands, a total of 16 textural variables were ultimately extracted: P_ME1_, P_ME2_; P_HO1_, P_HO2_; P_CO1_, P_CO2_; P_DI1_, P_DI2_; P_EN1_, P_EN2_; P_SE1_, P_SE2_; P_COR1_, P_COR2_.

#### 2.2.3. Acquisition and Processing of Topographic Data

Additionally, we extracted Digital Elevation Model (DEM), slope, and aspect data for use in analyzing the driving factors behind the spatial patterns of mangrove carbon stock. The DEM data employed in this study were sourced from Geospatial Data Cloud [21]. After undergoing projection transformation and resampling, the data were aligned with the GF-1 imagery in terms of spatial resolution and projected coordinate system. Slope and aspect data were derived from the processed DEM [22].

### 2.3. Construction of Biomass Estimation Models

#### 2.3.1. Modeling the Relationship Between AGB and Remote Sensing Variables

Based on the different mangrove species, the 129 samples were randomly divided into two independent datasets: a training set of 69 samples and a validation set of 60 samples. During the sample separation process, ensure spatial separation to reduce the risk of overly optimistic accuracy indicators caused by spatial autocorrelation of mangrove ecological data. A total of 24 variables, including band indices, vegetation indices, and texture features after dimensionality reduction, were extracted from the remote sensing imagery as independent variables for constructing the AGB estimation model. Due to issues of information redundancy, only a subset of these variables is useful in the AGB estimation model, making the identification of optimal variables a critical step. First, we used the Pearson correlation coefficient to assess the correlation between remote sensing variables and AGB. Only variables with statistically significant correlations were retained for AGB estimation.

A multiple linear regression analysis was conducted to establish the relationship model between AGB and remote sensing variables, generating a coefficient R to indicate their degree of correlation. The optimal model for estimating mangrove AGB was selected based on the highest R^2^ and the lowest RMSE. To achieve this, this study employed Pearson correlation at a 95% significance level. The general mathematical expressions for the multiple linear regression model, R^2^, and RMSE are shown in Equations (1)–(3) [23].

Y = β_0_ + β_1_X_1_ + β_2_X_2_ + β_3_X_3_ + … + β_k_X_k_ (1)

where Y is the dependent variable, X_1_, X_2_, X_3_, …, X_k_ are the independent variables, β_0_ is the constant term, and β_1_, β_2_, β_3_, …, βk are the partial regression coefficients.

(2) $$R^{2}\;=1-\frac{\sum_{i=1}^{n}{\left({\hat{y}}_{i}\;-\;y_{i}\right)}^{2}}{\sum_{i=1}^{n}{\left(y_{i}\;-\;\bar{y}\right)}^{2}}$$

(3) $$\mathrm{RMSE}=\sqrt{\frac{\sum_{i=1}^{n}{\left({\hat{y}}_{i}\;-\;y_{i}\right)}^{2}}{n}}$$

In the equations above: y is the observed value, $\bar{y}$ is the mean of the observed values, $\hat{y}$ is the predicted value, and n is the sample size.

Multiple linear regression was preferred over complex machine-learning methods in this study. Considering our field dataset, several mangrove species have relatively limited sample sizes, which cannot meet the large-sample requirement for stable training of most machine-learning models. Additionally, multiple linear regression can explicitly output correlation coefficients, enabling us to interpret the magnitude of correlation between each independent variable and AGB.

#### 2.3.2. Derivation of BGB Estimation Model Based on the AGB Estimation Model

A universal allometric relationship exists between plant AGB and BGB, which is commonly expressed as a functional relationship [24].

(4) $$\mathrm{BGB}=\beta X_{1}^{\alpha 1}X_{2}^{\alpha 2}\ldots {\;X}_{j}^{\alpha j}+\varepsilon$$

Among these, X_j_ represents the morphological characteristic variables of the plant’s aboveground parts, α_j_ and β are the model parameters, and ε denotes the error term.

Its general form is expressed as a univariate power function:

BGB = β*X*^α^ (5)

where X represents the AGB, α is the allometric coefficient, and β is the constant term. In practical applications, the power function allometric equation is often linearized via logarithmic transformation:

log_10_ BGB = α log_10_ X + log_10_ β (6)

log_10_ β represents the intercept of the line, and α denotes the slope. When α = 1, it indicates isometric growth between AGB and BGB; when α ≠ 1, it reflects allometric growth.

Based on the AGB and BGB data from species specific quadrats in this study, the data were log transformed. Using Standardized Major Axis (SMA) regression analysis implemented in R, allometric relationships between AGB and BGB for different mangrove species were established, ultimately leading to the development of BGB estimation models.

The regression parameters, statistical indicators and model coefficients of the AGB and BGB estimation models in this paper adopt a unified representation standard: all values are rounded to two decimal places.

#### 2.3.3. Model Accuracy Evaluation

For model accuracy evaluation, commonly used validation metrics—R^2^, RMSE, and mean relative accuracy (mAP)—were selected to assess the performance of the multiple regression model for predicting AGB and the derived BGB estimation model. Values of R^2^ and mAP closer to 1 and RMSE closer to 0 indicate higher predictive accuracy of the model. Additionally, the goodness of fit to the 1:1 line was used to test model accuracy, specifically to determine whether the estimated data and validation samples exhibited patterns of overestimation or underestimation. If the fitted line is biased toward the estimated values, overestimation occurs; if it is biased toward the field-measured values, underestimation occurs.

### 2.4. Mangrove Species Classification

To accurately estimate the biomass of different mangrove species, this study employed the Maximum Likelihood Classifier (MLC) for supervised classification in ENVI 5.3 software. MLC is a statistical-based image classification method that utilizes Bayesian theory to determine the probability of each pixel belonging to each category and assigns it to the class with the highest probability. The implementation steps mainly include the following stages:

(1) Selecting representative training areas for each mangrove species on the image based on field survey samples;
(2) Generating spectral signature files for each category from the corresponding training samples, characterized by mean vectors and covariance matrices;
(3) Using the MLC algorithm to calculate the membership probability of each pixel and as-signing it to the class with the highest probability;
(4) Producing the classification map and subsequently validating and adjusting the results.

### 2.5. Analysis of Driving Mechanisms for Mangrove Carbon Stock

#### 2.5.1. SHAP

We employed Shapley Additive Explanations—SHAP to interpret the final model. SHAP is a game theory-based method that interprets complex, “black-box” models by revealing the positive or negative influence and contribution of each feature to the prediction outcome [25]. The specific formula for SHAP feature contribution is as follows:

(7) $$\mathrm{SHAP}(i,j)\;=\;\Phi \;+\;\sum_{k\;=\;1}^{m}(a_{\mathrm{ii}}\frac{1}{(m\;-\;1,\;k\;-\;1)}\sum_{\subseteq M\backslash \left\{i\right\},\;\left|S\right|\;=\;k\;-\;1}(f(S\cup \left\{i\right\}\;-\;f(S)))$$

SHAP(i,j) represents the SHAP value of the i-th feature for the j-th sample; Φ is the baseline value, which is the average SHAP value of the model for all features; m is the total number of features; k is the number of features in the feature subset; S is a subset of features that does not include the i-th feature; f (S) is the model′s prediction value for sample j given the feature subset S; f(S∪{i}) is the model′s prediction value for sample j given the feature subset S plus the i-th feature; (m − 1, k − 1) is the number of combinations, which represents the number of combinations in which k − 1 features are selected from m − 1 features.

#### 2.5.2. Geographical Detector

The geographical detector is a suite of statistical methods designed to identify spatial heterogeneity and reveal its underlying driving factors [26]. Its core concept posits that if an independent variable significantly influences a dependent variable, their spatial distributions should closely resemble each other. In this study, two modules of the geographical detector—single factor detection and interaction detection—were employed to thoroughly investigate the effects of driving factors on the spatial heterogeneity of mangrove carbon stock.

(1) Single factor detection: This module assesses the extent to which a driving factor explains the spatial heterogeneity of mangrove carbon stock. It is quantified by the q value, expressed as:
  (8) $$q=1-\frac{\sum_{h=1}^{L}{N_{h}\sigma_{h}}^{2}}{{N\sigma }^{2}}$$
  where h = 1, …, L is the stratification, classification, or partition of variable Y or factor X; Nh and N are the number of units in layer h and the whole area respectively; σh2 and σ2 are the variances of mangrove carbon stock in layer h and the whole area respectively. The q-value ranges between [0, 1]. A higher value indicates stronger explanatory power of the driving factor X on carbon stock, and conversely.
(2) Interaction detection: This module examines whether the influences of different driving factors on carbon stocks are independent and evaluates whether the combined effect of factors X1 and X2 enhances or weakens their explanatory power on carbon stock [27]. First, the q values for the two driving factors—q(X1) and q(X2)—are calculated separately. Then, the interaction q value—q(X1∩X2)—is computed for the combined effect of the two factors. By comparing q(X1), q(X2), and q(X1∩X2), the interaction type can be categorized into five distinct classes (Table 3).

#### 2.5.3. Structural Equation Modeling

To investigate the direct and indirect pathways through which longitude, latitude, DEM, slope, and aspect influence mangrove carbon stock, this study constructed a structural equation model (SEM). SEM is a multivariate statistical method that integrates factor analysis and path analysis to test hypothesized causal relationships among variables [26]. First, an initial model was established, primarily describing the relationships between Lon, Lat, DEM, slope, aspect, and carbon stock. Subsequently, model fit was evaluated using the χ^2^ test, *p*-value, standardized root mean square residual (SRMR), comparative fit index (CFI), and goodness-of-fit index (GFI). All SEM analyses were conducted in SPSSAMOS 24.0.

## 3. Results

### 3.1. Results of Mangrove Species Classification

**Figure 3:**
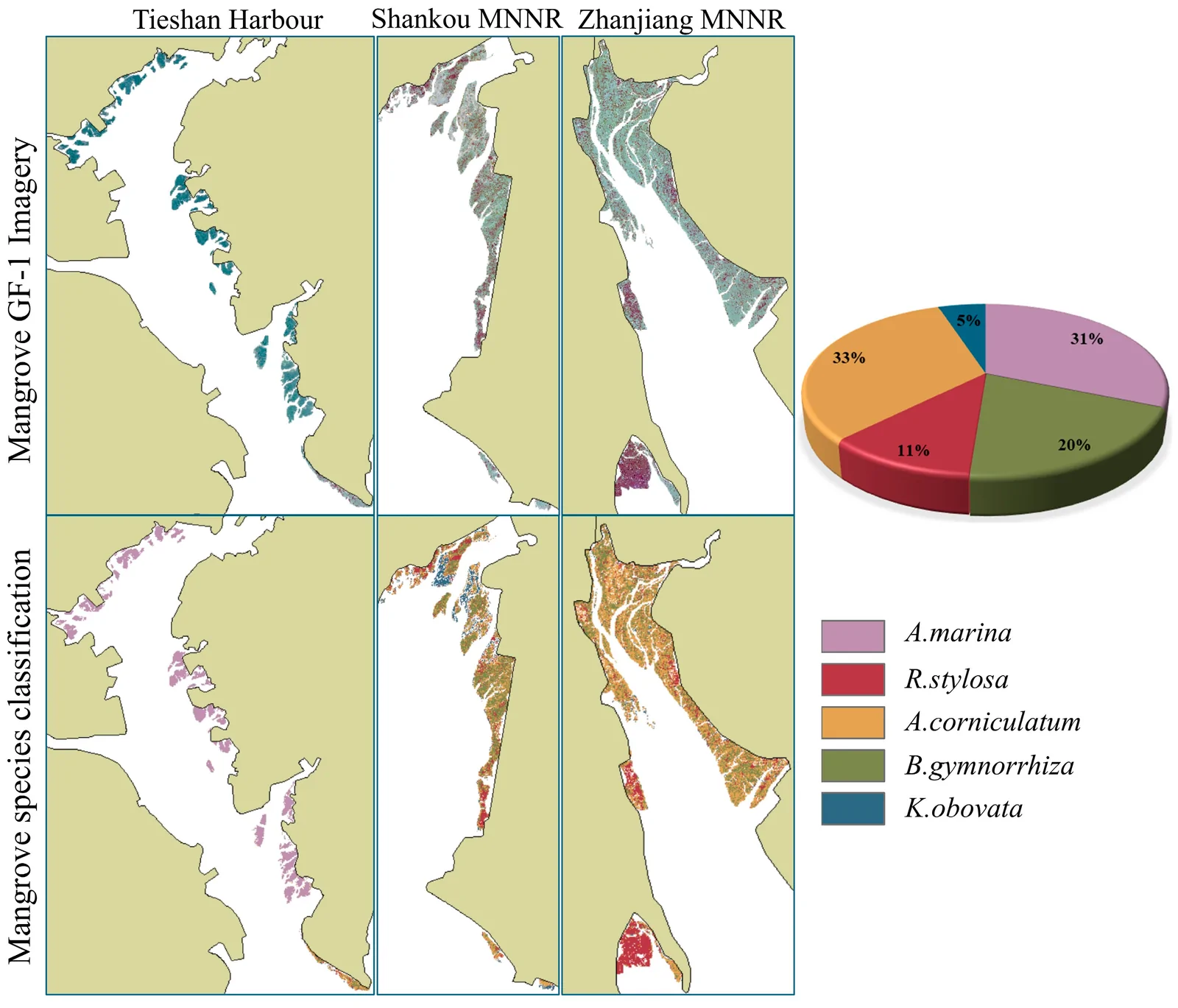
Mangrove species classification obtained by maximum likelihood method.

Using the MLC method and based on field-verified species data, the mangroves in the study area was classified into five species: *A. marina*, *B. gymnorrhiza*, *R. stylosa*, *A. corniculatum*, and *K. obovata*, accounting for 31%, 20%, 11%, 33%, and 5% of the total area, respectively. The classification result is shown in Figure 3. The accuracy of the mangrove species classification was assessed using a confusion matrix (Table 4). The overall accuracy reached 98.18%, with a Kappa coefficient of 0.94. The confusion matrix also indicated that user’s accuracy ranged from 90% to 98%, while producer’s accuracy varied between 85% and 99%. The lowest classification accuracy was observed for *B. gymnorrhiza*, with a producer′s accuracy of 89.72%. Misclassifications occurred mainly as *R. stylosa* (3.40%) and *A. corniculatum* (6.88%). The other four species achieved mapping accuracies above 90%, with *A. marina* reaching 100% and *R. stylosa* attaining 99.67%. Overall, the species classification in this study demonstrated high accuracy.

### 3.2. Inversion of Biomass and Carbon Stock at the Species Level

#### 3.2.1. Species-Specific Biomass Estimation Models

The AGB estimation models for different mangrove species based on vegetation index and texture are presented in Table 5. The sample sizes of training-set for *A. marina*, *B. gymnorrhiza*, *R. stylosa*, *A. corniculatum*, and *K. obovata* were 18, 12, 5, 22, and 12, respectively. Durbin-Watson (D-W) statistics were applied to test residual autocorrelation for these species-specific regression models. All D-W values fall within the reasonable range of 1.5–2.5, indicating no significant residual autocorrelation and demonstrating that the regression models satisfy the required statistical assumptions. Vegetation indices and texture features, as modeling variables, exhibit marked differences in the AGB estimation models across different species. For instance, the *A. marina* estimation model relies primarily on vegetation indices, while that of *K. obovata* is dominated by texture features. This distinction mainly stems from structural heterogeneity and spectral variations among mangrove species. *A. marina* has thicker leaves with high near-infrared reflectance, leading to elevated vegetation index values—especially NDVI. Its canopy structure is relatively uniform and simple, resulting in weaker texture expression. In contrast, *K. obovata* possesses a dense, undulating, and structurally complex canopy, which manifests as rich texture variation in remote sensing imagery. Notably, for the *R. stylosa* model, there is an obvious decrease from R^2^ (0.780) to adjusted R^2^ (0.548). This considerable drop is largely associated with its limited training sample size relative to the number of predictor variables, even though the model passed statistical diagnostic tests. These findings indicate that adopting differentiated remote sensing factors for biomass estimation models is key to enhancing model accuracy and reliability.

**Figure 4:**
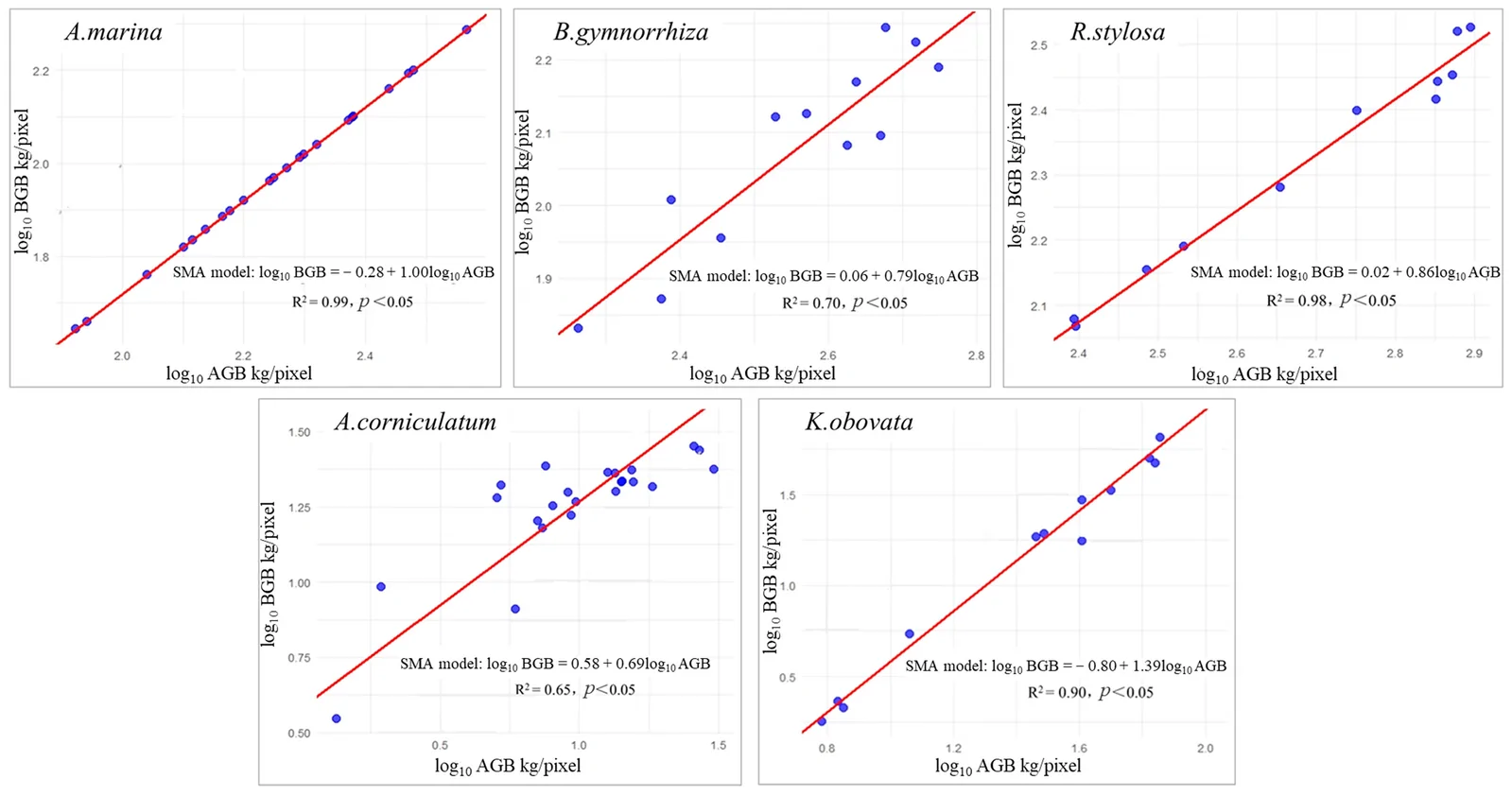
Allometric relationship between AGB and BGB of different mangrove species based on SMA method.

Using the SMA method, the allometric relationships between AGB and BGB for the five mangrove species were analyzed (Figure 4). The R^2^ values of the models for each species ranged from 0.65 to 0.99, indicating an overall good fit. Furthermore, the test for isometric growth was significant (*p* < 0.05) for all species, confirming a clear allometric relationship between AGB and BGB. Based on the previously developed AGB estimation models and these allometric relationships, the final BGB estimation models were derived as summarized in Table 6.

**Figure 5:**
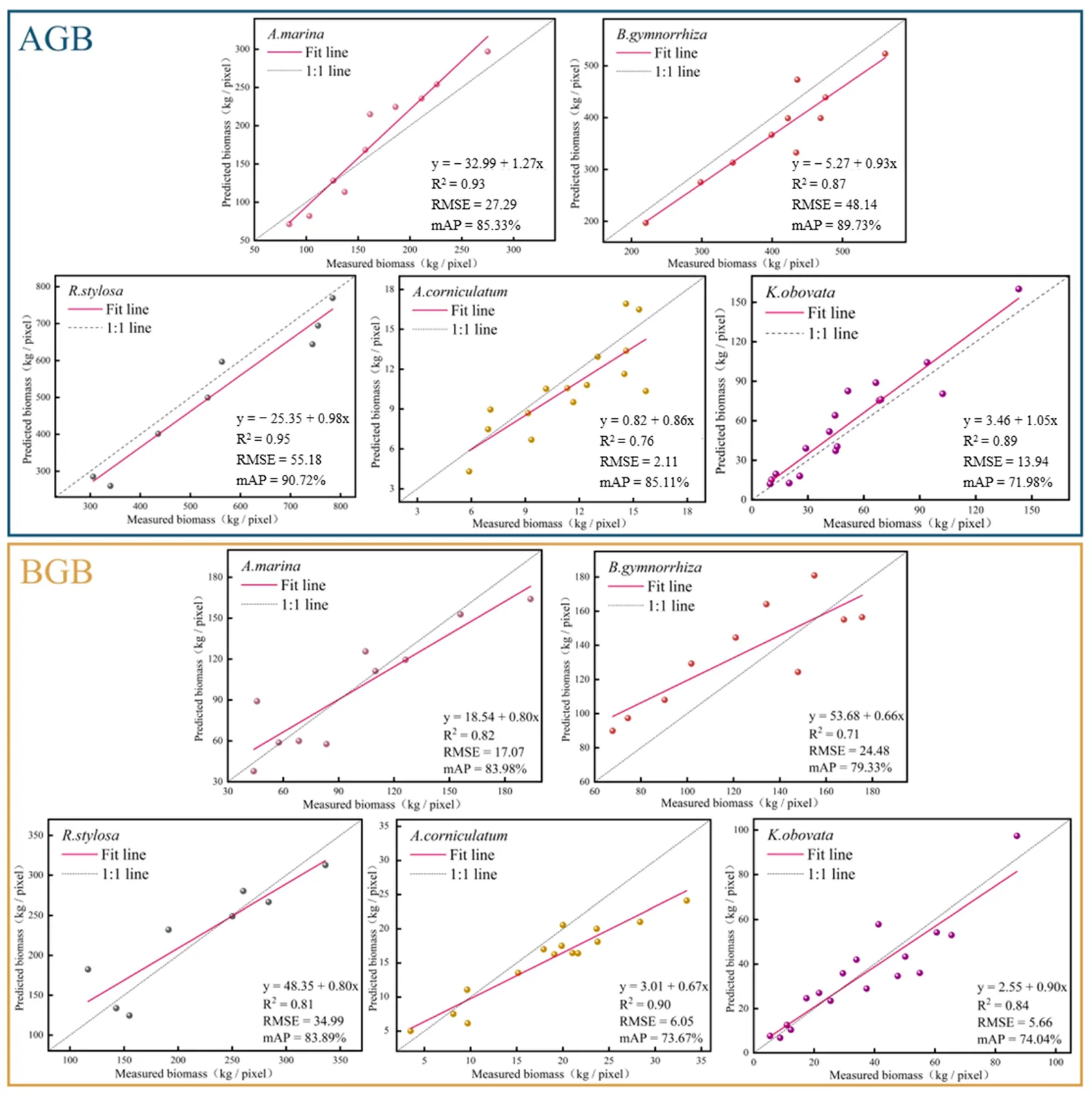
Accuracy assessment of estimation models for AGB and BGB of different mangrove species.

Based on the validation dataset from field surveys, the accuracy of the AGB and BGB estimation models for different mangrove species was evaluated. The sample sizes of validation-set for *A. marina*, *B. gymnorrhiza*, *R. stylosa*, *A. corniculatum*, and *K. obovata* were 10, 10, 8, 15, and 17, respectively. The accuracy assessment results (Figure 5) show that the models achieved high precision in estimating both AGB and BGB across species (R^2^ ≥ 0.71), with mAP values all above 70%. Examining the goodness of fit against the 1:1 line, each species exhibited a distinct distribution pattern. In AGB estimation, *K. obovata* showed a tendency to be overestimated, while *B. gymnorrhiza* and *R. stylosa* were at risk of underestimation. Overall, most scattered points clustered closely around the diagonal, and the regression line between measured biomass and predicted biomass aligned well with the 1:1 trend, indicating good model performance.

#### 3.2.2. Spatial Distribution of Biomass and Carbon Stock

In this study, the derived AGB and BGB estimation models were applied to each pixel of the GF-1 imagery to estimate mangrove biomass across the entire image coverage. Subsequently, mangrove carbon stock was calculated based on species-specific biomass-to-carbon conversion factors. The carbon conversion coefficients for AGB of the five species—*A. marina*, *B. gymnorrhiza*, *R. stylosa*, *A. corniculatum*, and *K. obovata*—were set as 0.428 [28], 0.463 [29], 0.49 [30], 0.44 [28], and 0.448 [28], respectively. The BGB carbon conversion coefficient used was 0.39 [31].

**Figure 6:**
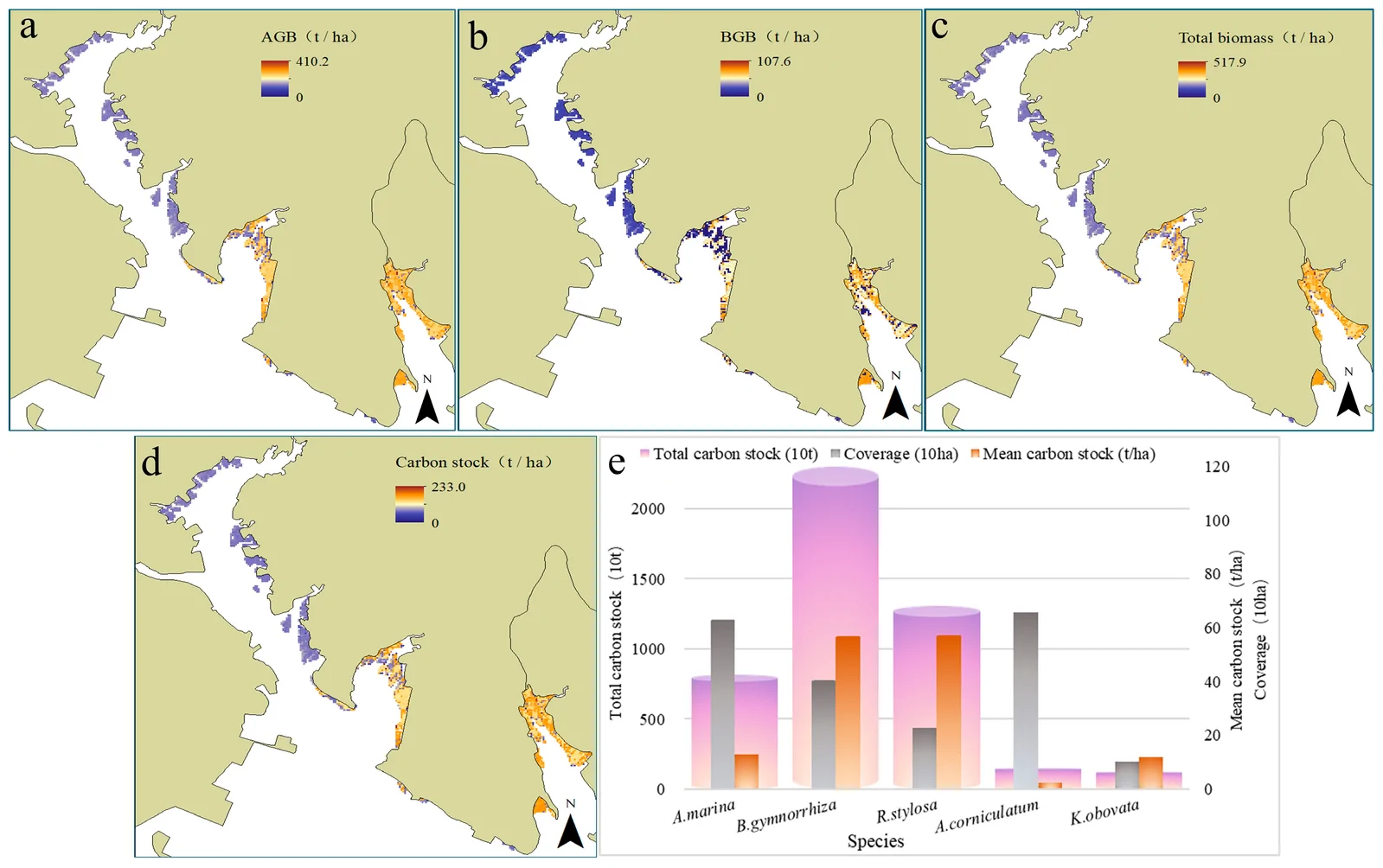
Spatial distribution of AGB (**a**), BGB (**b**), total biomass (**c**), carbon stock (**d**) of mangrove, and differences in carbon stock among species (**e**).

In the study area, the estimated AGB of mangroves ranges from 0.21 to 410.2 t/ha (Figure 6a), with a mean value of 41.31 t/ha. The BGB is estimated between 0.54 and 107.65 t/ha (Figure 6b), averaging 19.67 t/ha. Total biomass varies from 0.75 to 517.81 t/ha (Figure 6c), with a mean of 60.97 t/ha. It should be noted that these extremely high biomass values correspond to only a very small number of pixels. Such upper bounds may originate from outlier edge-pixels and residual image noise in pixel-wise inversion, although locally mature mangrove communities can also yield relatively high biomass estimates. Carbon stock per pixel ranges from 0.10 to 767.70 t/ha (Figure 6d), with an average of 23.18 t/ha. The sum of pixel values indicates a total carbon stock of 46,871.76 t for the entire study area. Biomass and carbon stock levels in Tieshan Harbor are significantly lower than those in Shankou MNNR and Zhanjiang MNNR. High-value areas are concentrated in a narrow offshore strip within Zhanjiang MNNR and show a decreasing trend toward the nearshore zone. The spatial distribution of carbon stock closely corresponds to that of total biomass. Figure 6e illustrates notable differences in total carbon stock, coverage, and mean carbon stock across mangrove species. Total carbon stock is derived from the product of mean carbon stock and species coverage. *B. gymnorrhiza* and *R. stylosa* exhibit similarly high mean carbon stock, which can be attributed to their tall stature, thick trunks and dense branching that greatly improve individual biomass and unit-area carbon density; however, due to its larger coverage, *B. gymnorrhiza* achieves the highest total carbon stock. Although *A. marina* has greater coverage than both *B. gymnorrhiza* and *R. stylosa*, its total carbon stock is lower owing to its thin foliage and relatively sparse canopy structure, indicating a comparatively weaker carbon storage capacity. *A. corniculatum* is categorized as dwarf shrub mangrove with inherently low single-plant biomass, so it shows an extremely low mean carbon stock, and despite having the widest coverage, its total carbon stock remains limited. *K. obovata* displays both low mean carbon stock and limited coverage, constrained by narrow spatial distribution and weak individual carbon sequestration performance, resulting in the lowest total carbon stock among the species.

### 3.3. Spatial Heterogeneity and Driving Mechanisms of Carbon Stock

**Figure 7:**
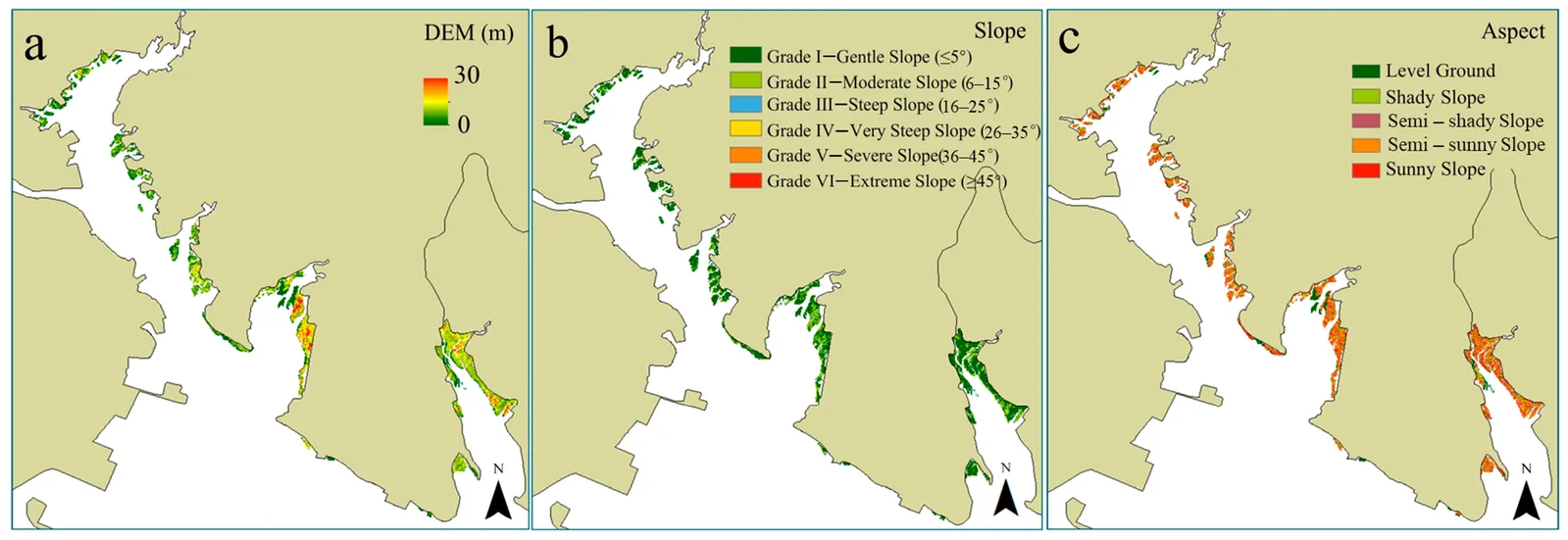
Spatial distribution of DEM (**a**), slope (**b**), and aspect (**c**) in the study area.

We considered five variables—Lon, Lat, DEM, slope, and aspect—as driving factors, which were treated as gridded data influencing mangrove carbon stock. Figure 7 presents the spatial distribution maps of DEM, slope, and aspect across the mangrove study area. To more clearly reveal their influence on carbon stock at different levels, slope and aspect values in this study were categorized into classes.

#### 3.3.1. Pearson Correlation

**Figure 8:**
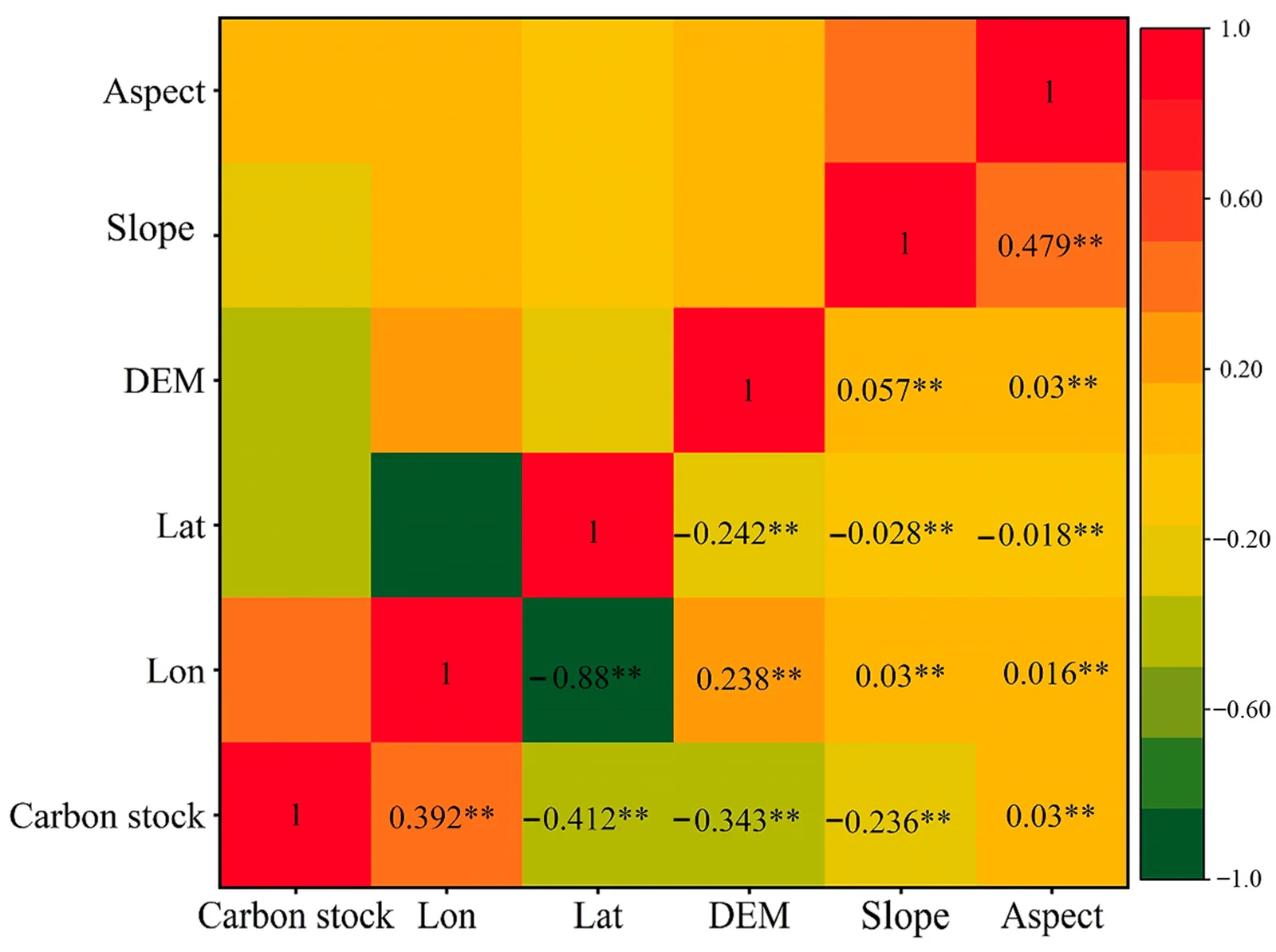
Pearson correlation between carbon stock and driving factors (Note: ** indicates significance at *p* < 0.01).

The Pearson correlation coefficients between each driving factor and carbon stock are shown in Figure 8. Carbon stock exhibited a significant positive correlation with Lon and significant negative correlations with Lat, DEM, and slope. These relationships suggest a potential decreasing trend in carbon stock from the southwest to the northeast of the study area, as well as a clear declining trend with increasing DEM and slope values. In contrast, aspect showed a weak correlation with carbon stock, indicating a relatively minor influence. The ranking of the factors based on their correlation strength with carbon stock was as follows: Lat > Lon > DEM > slope > aspect.

#### 3.3.2. SHAP

**Figure 9:**
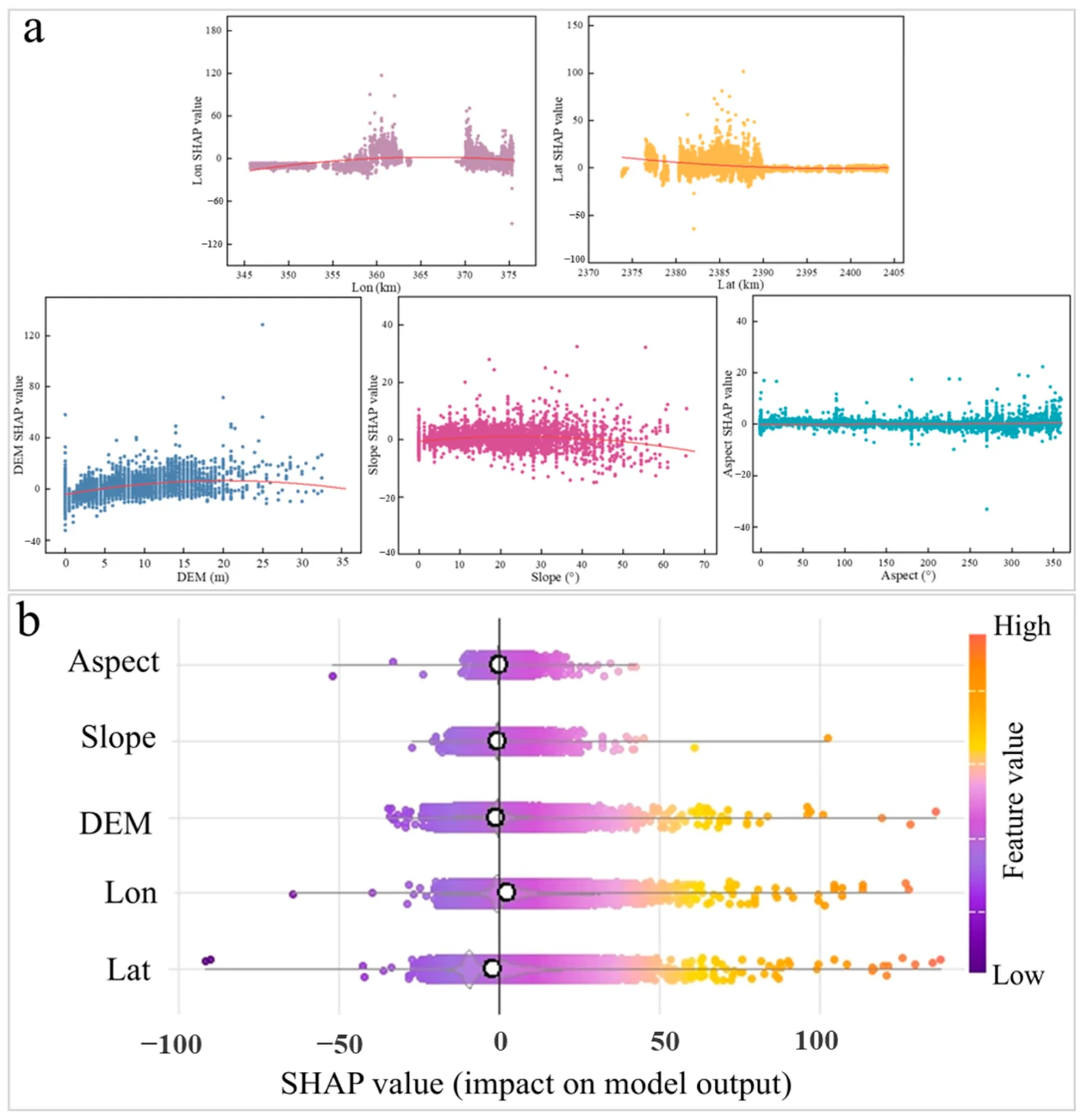
SHAP feature dependency (**a**) and contribution plot (**b**).

Carbon stock was obtained by converting total biomass calculated from the developed species-specific AGB and BGB estimation models. The SHAP method was then used to quantify the contribution of each driving predictor to these carbon-stock values. The SHAP method was employed to assess the contribution of each driving feature at every sample point to the model output. In the feature dependence plots, a larger fluctuation range of SHAP values indicates a stronger influence of that feature on carbon stock (Figure 9a). Significant differences were observed in the impact patterns among the driving factors: Lon exhibited a bidirectional effect on carbon stock, with the fitted curve remaining generally flat. Within the 360–375 km range, SHAP values were predominantly positive and densely distributed, suggesting a relatively significant promoting effect on carbon accumulation. In contrast, within the 345–360 km range, values were mostly negative, indicating a certain inhibitory effect; Lat showed SHAP values shifting from positive to negative. Lower latitudes were generally associated with higher positive values, implying that warmer temperatures and more abundant precipitation at these latitudes enhance vegetation carbon sequestration. In contrast, higher latitudes tended to suppress carbon fixation; DEM displayed a unimodal pattern in its SHAP curve, rising initially and then declining. Below 15 m, SHAP values increased with elevation, reflecting that wetter conditions in low-DEM areas support higher vegetation productivity and thus promote carbon stock. Beyond 20 m, SHAP values gradually decreased and turned negative, inhibiting carbon accumulation. Slope showed SHAP values concentrated mainly in the negative range, with a slightly downward-sloping fitted line. This indicates an overall inhibitory effect of slope on carbon stock, particularly in steeper areas where SHAP values deviate more strongly in the negative direction. This is likely related to severe soil erosion and difficulties in vegetation establishment, leading to poor carbon sequestration capacity; Aspect had SHAP values uniformly distributed around zero, with a nearly horizontal fitted line, suggesting a weak influence on carbon stock.

Figure 9b presents the global summary plot of the ensemble model, where the importance of each feature is ranked from bottom to top. The ranking result aligns with that derived from Pearson correlation analysis. Lon, Lat, and DEM contribute significantly to mangrove carbon stock, while slope and aspect exhibit relatively minor influences. The plot also reveals the direction of feature contributions. All features span both negative and positive SHAP values, indicating that depending on their specific values, each feature can either positively (SHAP > 0) or negatively (SHAP < 0) affect the model output, reflecting their bidirectional influence. In terms of average contribution, Lon shows a positive mean SHAP value (white circle positioned to the right of zero), suggesting an overall positive driving effect on the model output. In contrast, Lat and DEM exhibit negative mean SHAP values, indicating an overall negative driving effect. Slope and aspect, with smaller mean absolute SHAP values, demonstrate relatively weaker impacts.

#### 3.3.3. Geographical Detector

**Figure 10:**
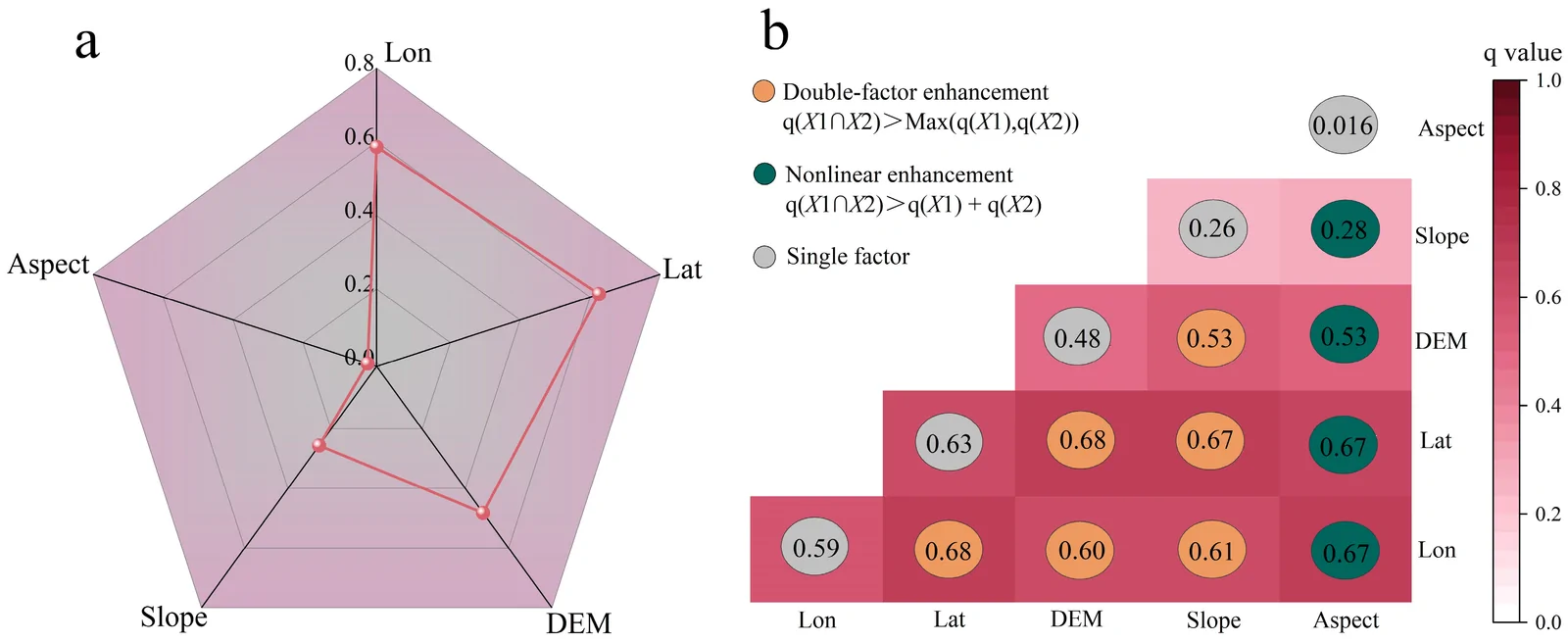
Drivers of spatial heterogeneity in mangrove carbon stock: (**a**) Single-factor detection, (**b**) Interaction detection.

The single factor detection results reveal the extent of influence of different features on carbon stock (Figure 10a). The explanatory power of the five detected factors for the spatial heterogeneity of carbon stock is ranked as follows: Lat (0.63) > Lon (0.59) > DEM (0.48) > Slope (0.26) > Aspect (0.016). This ranking aligns with those derived from Pearson correlation and SHAP feature importance analysis. Among them, Lon and Lat dominate the explanation of spatial variation in carbon stock, highlighting the constraining effect of thermal zones on vegetation types and carbon sequestration capacity. DEM acts as a secondary dominant factor, creating vertical ecological gradients through the redistribution of water and heat, thereby leading to heterogeneous carbon accumulation rates and stocks across different elevations in mangrove ecosystems. Slope shows weaker explanatory power, while aspect contributes negligibly. These findings indicate that the spatial pattern of carbon stock is primarily controlled by macro scale geographic and climatic patterns, with relatively minor modification by local topographic factors.

Figure 10b illustrates the interactive effects of any two driving factors on carbon stock. As shown, the interaction between any two factors yields a higher q value than that of each corresponding single factor. There are no cases of mutual weakening or independence, indicating that these factors do not act in isolation but jointly drive the spatial heterogeneity of carbon stock through synergistic interactions. Two types of interaction effects are observed. Compared with double factor enhancement, nonlinear enhancement leads to a more pronounced increase in the interactive influence of the two factors. Although aspect alone has a relatively small effect on the spatial variation of carbon stock, its influence increases significantly when interacting with other factors—particularly with Lon and Lat, where the explanatory power is strongest. Among all factor interactions, combinations involving Lon and Lat consistently yield high q values above 0.6, underscoring that the interaction between latitudinal longitudinal and topographic factors constitutes the core geographical process regulating the spatial heterogeneity of carbon stock, jointly shaping the potential for vegetation growth and carbon stock.

#### 3.3.4. SEM

**Figure 11:**
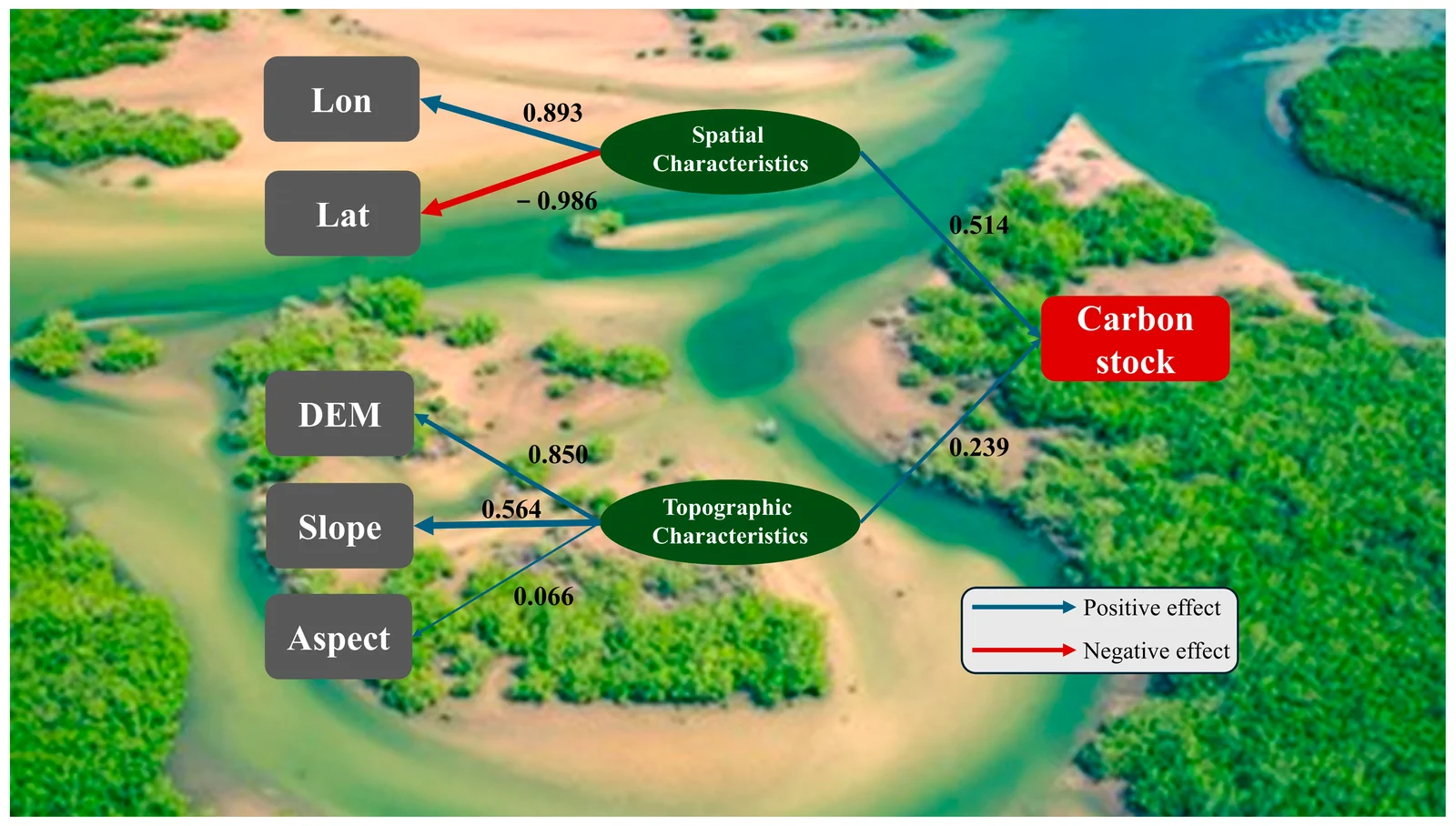
SEM of driving factors and carbon stocks.

Lon and Lat were classified as spatial characteristics, while DEM, slope, and aspect were grouped as topographic characteristics, serving as latent variables to analyze their impacts on mangrove carbon stock in the study area (Figure 11). The SEM results show that all path relationships are significant at the *p* < 0.001 level. The absolute values of the path coefficients for Lon (0.893) and Lat (−0.986) are both close to 1, and together as spatial characteristics they explain 0.514 of the variance in carbon stock. In contrast, DEM (0.850) and slope (0.564) strongly contribute to topographic characteristics, whereas aspect shows only a weak path coefficient (0.066) and limited explanatory power. Topographic characteristics collectively explain 0.239 of the variance in carbon stock. These findings confirm that spatial characteristics, represented by Lon and Lat, are the core regulatory factors of carbon stock, while topographic characteristics play a relatively weaker driving role. This result aligns with the interaction effects identified by the geographical detector.

Table 7 presents the overall goodness-of-fit indices for the structural equation model. The χ^2^/DF value was 8, exceeding the commonly-used acceptable threshold of 5. It should be noted that the chi-square statistic is highly sensitive to sample size. For large sample sizes, trivial practical discrepancies between model and observations can produce large χ^2^ and inflated χ^2^/DF values. For large-sample scenarios, we should not categorically reject the model merely because of a significant chi-square value or χ^2^/DF > 5. Comprehensive evaluation should be conducted by combining multiple indicators such as RMSEA, CFI, and TLI. A model can still be considered acceptable even with χ^2^/DF = 8 when RMSEA < 0.08, particularly under large-sample conditions. CFI and TLI values greater than 0.90 indicate considerable improvement relative to the worst-case null independent model, implying potential practical value of the constructed model.

In addition, the PGFI value was 0.372, which did not reach the threshold of 0.5, suggesting room for improvement in model parsimony. Nevertheless, the model can still be regarded as acceptable if other major fit indices satisfy the criteria of CFI > 0.92 and RMSEA < 0.07. In our case, CFI = 0.961 and RMSEA = 0.065. These fit indices demonstrate favorable overall fitness for the structural relationship between mangrove carbon stock and its driving factors, and the model can effectively interpret the path mechanisms among variables.

## 4. Discussion

### 4.1. Necessity of High-Precision Species Classification and Species-Specific Modeling Research

The development of species-specific biomass estimation models represents an innovative aspect of this study that overcomes the limitations of conventional modeling approaches. Existing research often treats mangrove communities as homogeneous entities and applies generalized models, leading to significant errors in areas with complex species composition [32]. This study reveals notable differences in the biomass related predictor variables among different mangrove species: *A. marina* relies on vegetation indices (NDVI, EVI, SAVI), while *K. obovata*, with its dense and structurally complex canopy, is better characterized by texture features, which more effectively reflect variations in biomass accumulation. In terms of model accuracy, the R^2^ values for both AGB and BGB estimation models across all species exceeded 0.71. Compared with the constructed unified generalized model (R^2^ = 0.60) [33], fitting effect was better, but there is still room for further improvement in accuracy. These results verify the effectiveness of the specific species modeling strategy in reducing estimation errors compared with the unified modeling.

The allometric relationship between AGB and BGB established using the SMA method enables the indirect and accurate inversion of BGB, addressing the challenge that remote sensing technology faces in directly detecting belowground vegetation carbon pools. Compared to studies that derive BGB models based on a fixed ratio between AGB and BGB [24,34] the allometric relationship model constructed in this study through the SMA method fully captures the dynamic relationship between AGB and BGB across different mangrove species, as well as interspecific differences. This effectively avoids the estimation bias caused by ignoring species heterogeneity and growth stages in the fixed-ratio approach, significantly improving the accuracy of BGB inversion. By coupling the estimation of AGB and BGB, both aboveground and belowground components of the mangrove vegetation carbon pool are comprehensively covered. This provides data support for a holistic assessment of carbon stock and addresses the common limitation in many studies that focus only on AGB while neglecting the contribution of the belowground carbon pool [35,36].

The high-precision classification of five mangrove species in the study area was achieved using the MLC, with an overall accuracy of 98.18% and a Kappa coefficient of 0.94. This accuracy surpasses that of species classification results derived from medium-resolution imagery. For instance, mapping mangrove species along the southern Chinese coast solely with 10 m Sentinel-2 time-series data yielded an overall accuracy of 84% [37]. Even when integrating GF-1 optical, Sentinel-2 and SAR multi-source features for mangrove species discrimination in subtropical coastal Guangdong, the overall classification accuracy reached only 90.13% [38]. The improved classification accuracy in this study can be attributed to two main factors: first, the high spatial resolution (2 m) of the fused GF-1 imagery enabled clear capture of canopy structural differences among mangrove species, particularly enhancing the distinction between species with pronounced canopy texture, such as *K. obovata*, and those with homogeneous structure, like A. marina; second, precise field survey data allowed for refined classification thresholds, effectively reducing misclassification errors.

It should be noted that the allometric equations adopted in this work are existing regional models rather than locally calibrated using destructive samples in the study area. These equations comply with China’s mangrove carbon survey standard and have been widely applied in subtropical mangroves of Guangdong and Guangxi [39,40]. Constrained by conservation regulations, destructive sampling is difficult to implement in nature reserves to develop site-specific models.

### 4.2. Analysis of Spatial Distribution Characteristics and Regional Variations in Carbon Stock

The total carbon stock of mangroves in the study area is 46,871.76 t, with an average carbon density of 23.18 t/ha. This value is slightly higher than the mean total vegetation-carbon density (18.1 t/ha) for fragmented mangrove stands along Guangxi coastal zones [41], while it is lower than the range (30.1–49.4 t/ha) reported for well-developed mangrove communities in Guangdong coastal nature reserves [42]. This discrepancy is attributed to the higher species richness of mangroves in Hainan Island (27 species), which enhances the carbon accumulation capacity of the community [43]. Furthermore, regional habitat and anthropogenic disturbance may also affect the comparability of the results. In terms of spatial pattern, areas with high carbon stock are concentrated in an offshore narrow strip within the Zhanjiang MNNR, while low values are distributed in Tieshan Harbor. This distribution pattern is jointly regulated by multiple factors.

Species composition differences serve as a core biological factor driving the spatial heterogeneity of carbon stock. *B. gymnorrhiza*, with its relatively high average carbon density and extensive distribution area, contributes the highest total carbon stock. In contrast, although *A. corniculatum* has the widest distribution, it’s extremely low average carbon density limits its overall contribution to total carbon stock. The synergistic effect between the carbon density of dominant species and their distribution area determines the regional total carbon stock level. Furthermore, while *R. stylosa* exhibits a carbon density comparable to that of *B. gymnorrhiza*, its limited distribution area constrains its contribution to the total carbon stock. This finding suggests that, in mangrove carbon sink conservation efforts, attention should not only be given to high-carbon-density species but also to the maintenance of species distribution patterns.

Regional habitat and anthropogenic disturbance differences further intensify the spatial heterogeneity of carbon stock. As a port area, Tieshan Harbor experiences significant anthropogenic disturbances such as shipping and reclamation activities, which notably suppress the growth of nearshore mangroves and result in lower carbon stock levels. In contrast, the offshore zones of Shankou and Zhanjiang MNNR are less disturbed, with stable hydrodynamic conditions and sufficient soil fertility, providing a favorable environment for mangrove growth and forming areas of high carbon stock. This phenomenon indicates that anthropogenic disturbance is also a key driving factor influencing carbon stock, highlighting the critical role of protected areas in maintaining the carbon sequestration function of mangroves [33].

### 4.3. Driving Mechanisms of Spatial Patterns in Carbon Stock

This study comprehensively employed Pearson correlation, SHAP, geographical detector, and SEM to investigate the driving mechanisms, spanning from single factor effects and interactions to latent variable path analysis. The results revealed that the importance ranking of the driving factors is Lat > Lon > DEM > slope > aspect. Furthermore, geospatial factors (Lon and Lat) play a dominant role, while topographic factors (DEM, slope, aspect) serve complementary and synergistic roles, with significant interactions existing among these factors. These findings deepen the theoretical understanding of the driving mechanisms behind mangrove carbon stock.

Notably, Lon and Lat are essentially spatial coordinate indicators rather than direct ecological factors, and their dominant driving effects on carbon stock are realized by mediating core environmental and ecological gradients including temperature, precipitation, seawater salinity, and coastal hydrological conditions. Lat dominates the spatial differentiation of regional temperature and precipitation conditions, showing a significant negative correlation with mangrove carbon stock. Lower-Lat regions possess higher annual average temperature and more sufficient precipitation, which significantly improve mangrove photosynthetic efficiency and primary productivity, accelerate vegetation biomass accumulation, and promote conditions burial and sequestration of sediment organic carbon [44]. In contrast, high-Lat areas are restricted by low temperature and insufficient hydrothermal supply, which inhibits mangrove growth and carbon accumulation capacity, consistent with the global latitudinal pattern of mangrove blue carbon distribution [45]. Lon affects carbon stock patterns by regulating coastal land–sea gradients, regional salinity distribution, and tidal hydrodynamic stability. In the 360–375 km offshore range far from terrestrial interference, the tidal flat environment is stable with moderate and constant salinity, reduced tidal fluctuation, and almost no anthropogenic disturbance, creating optimal hydro-sediment conditions for mangrove carbon accumulation. However, the 345–360 km nearshore zone is affected by frequent terrestrial material input and human activities, resulting in drastic salinity fluctuations and unstable tidal flat habitats, which suppress mangrove growth and reduce soil organic carbon stock.

Among topographic factors, DEM acts as a key local regulatory factor by reshaping microscale hydrothermal distribution, soil moisture conditions, and sediment accumulation environments. In low DEM areas below 15 m, the terrain is flat, water and humidity conditions are superior, sediment accumulation is continuous and stable, and vegetation productivity is maintained at a high level, thus forming high-carbon stock mangrove habitats [46]. As elevation rises above 20 m, reduced groundwater recharge and surface water retention lead to insufficient soil water supply and weakened mangrove adaptability, significantly inhibiting carbon accumulation. Slope presents a significant negative driving effect; steep slopes easily trigger soil erosion and sediment loss, damage mangrove root systems and growth habitats, hinder vegetation colonization and community stability, and ultimately reduce ecosystem carbon sequestration capacity [33]. Aspect only produces a marginal effect on carbon stock spatial patterns, with no dominant regulatory role in regional-scale mangrove carbon differentiation.

The geographical detector results verified strong synergistic interactions among all driving factors, and the interaction explanatory power of Lat and Lon with topographic factors all exceeded 0.6. This fully demonstrates that the spatial pattern of mangrove carbon stock is not governed by individual spatial or topographic factors, but arises from the coupled effects of large-scale hydrothermal spatial gradients and localized topographic habitat regulation [47]. The SEM results further quantified the difference in latent variable driving effects: the explanatory power of macroscale spatial characteristics (Lon and Lat, 0.514) was far higher than that of local topographic characteristics (0.239), confirming that large-scale geographical environmental gradients are the core mechanism dominating mangrove carbon stock spatial heterogeneity [48].

In summary, the spatial differentiation of mangrove carbon stock is driven by the multi-factor coupling of macro climatic-hydrothermal conditions (temperature, precipitation mediated by latitude and longitude), local topographic habitats, and human disturbance. The dominant role of spatial location essentially reflects the spatial differentiation of regional natural background conditions, while topographic factors play a local regulatory role, and human activities act as an important interference and modifying factor. Subsequent studies can integrate climatic factors (precipitation, temperature) and anthropogenic disturbance intensity to construct a more comprehensive driving factor system, further reveal the interactive coupling mechanism of natural and human factors on mangrove carbon stock, and provide more accurate theoretical support for regional mangrove blue carbon protection and carbon sink enhancement.

### 4.4. Research Limitations and Future Prospects

While this study has made progress in estimating mangrove carbon stock and understanding their driving mechanisms, several obvious limitations remain. First, the research area is limited to typical coastal regions of southern Guangxi, and the generalizability of the results requires validation on larger scales. Second, the sample size of mangrove species is not large enough. Although their models have passed statistical diagnosis, the limited sample size may still introduce potential overfitting risks for species-specific biomass estimation. Moreover, the temporal mismatch between field survey and remote-sensing image acquisition may bring additional uncertainties to the inversion results. In addition, despite high classification accuracy, errors from species classification will propagate and further affect carbon stock estimation. Third, this study only relies on optical satellite spectral and texture features for pixel-wise biomass inversion, without incorporating dendrometric parameters (tree height, diameter at breast height) as predictive variables. These metrics can only be obtained at discrete field sampling plots and cannot be acquired for every image pixel. This directly limits the explanatory power of the model, making it impossible to explain a certain proportion of biomass variance. Fourth, we did not include continuous tidal fluctuation data and soil physicochemical indicators into the driving factor and modeling system, which further fails to fully capture environmental interference on mangrove carbon accumulation.

Future research could proceed in two directions: first, expanding the study scope and validating the transferability of the developed models across wider mangrove areas and integrating Unmanned Aerial Vehicle (UAV), Synthetic Aperture Radar (SAR) or multi-spectral image fusion techniques to extract spatially continuous canopy structural information equivalent to dendromeric traits, further optimize species classification and biomass estimation models, mitigate sample-size-related risks and minimize uncertainties induced by temporal mismatch and classification errors, and reduce unexplained model variance; second, deepening the study of driving mechanisms by incorporating factors such as tree height, diameter at breast height, tidal cycles, and soil physicochemical properties to construct a more comprehensive system of driving factors. This would provide more precise scientific support for the refined management of mangrove blue carbon ecosystems.

## 5. Conclusions

This study developed a systematic analytical framework integrating species-specific biomass modeling, high-precision classification, and multi-dimensional mechanism analysis, which enabled high precision mapping and causal interpretation of spatial carbon stock patterns in typical mangrove areas. The main conclusions are as follows:

(1) Species-specific modeling significantly improved the accuracy of carbon stock estimation. By developing species-differentiated AGB remote sensing estimation models and coupling them with BGB estimation derived from allometric relationships, a complete assessment of mangrove vegetation carbon pools was achieved, with satisfactory validation accuracy (R^2^ for both AGB and BGB models exceeded 0.76).
(2) Species classification based on high-resolution imagery achieved high accuracy. Using GF-1 fused imagery at 2 m resolution, the overall classification accuracy reached 98.18%, providing reliable local data for subsequent analyses. The study area has an average mangrove carbon density of 23.18 t/ha and a total vegetation carbon stock of 46,871.76 t, exhibiting obvious spatial heterogeneity.
(3) The ranking of driving factors influencing mangrove carbon stock heterogeneity was Lat > Lon > DEM > slope > aspect, and widespread synergistic enhancement effects were observed among these factors. Specifically, macroscale spatial factors (Lat and Lon) are the dominant driving factors, jointly explaining 51.4% of carbon stock variation by regulating regional hydrothermal conditions and coastal hydrological gradients; local topographic factors (DEM, slope, aspect) play a supplementary modifying role, explaining 23.9% of the variance.

The high-resolution carbon stock distribution maps generated in this study, along with the clarified driving mechanisms, provide a solid scientific basis and data support for the precise management of mangrove protected areas, scientific planning of ecological restoration projects, and the development and monitoring of blue carbon initiatives.

This localized analytical framework can be extended to other subtropical mangrove wetlands in future research. Subsequent work will expand the research scope, integrate multi-source remote sensing data and environmental variables to further optimize model accuracy and enhance the generalizability of carbon estimation methods.

## Figures and Tables

**Table 1:** Allometric growth equations of mangrove in research area.

Species	AGB (kg)	BGB (kg)
*A. marina*	0.076123 × (D^2^H) − 0.222424	0.040168 × (D^2^H) − 0.12623
*B. gymnorrhiza*	0.186 × D^2.31^	0.0188 × (D^2^H)^0.909^
*R. stylosa*	0.2206 × D^2.4292^	0.261 × D^1.86^
*A. corniculatum*	0.02039 × (D^2^H)0.83739	0.199 × ρ^0.899^ × D^2.22^
*K. obovata*	0.05698 × D^2^ − 0.295595	0.009685 × (D^2^H) + 0.108358

D: diameter at breast height (cm); H: height (m); ρ: wood density (g/cm^3^).

**Table 2:** The remote sensing variable derived from GF-1 data for the estimation of mangrove AGB (Index formulas are general and not exclusive to GF-1 imagery).

Variable Type	Variable Name	Variable Description and Formulas	References
Band indices	Blue	B1	[13]
	Green	B2	
	Red	B3	
	Near-Infrared	B4	
Vegetation indices	NDVI	$\frac{B4\;-\;B3}{B4+B3}$	[14]
	SAVI	$\frac{(1+L)(B4\;-\;B3)}{B4+B3+0.5}$	[15]
	EVI	$\frac{2.5(B4\;-\;B3)}{B4+6B3\;-\;7.5B1+1}$	[16]
	DVI	B4 − B3	[17]
	RVI	$\frac{B4}{B3}$	[17]
	GNDVI	$\frac{B4\;-\;B2}{B4+B2}$	[18]
Texture features	ME, VA, HO, CO, DI, EN, SE, COR	Texture features of B1~B4	[19]

Note: L = 0.5 in most conditions.

**Table 3:** Interaction types of the geographical detector.

Range of q-Value	Interaction Types
q(X1∩X2) < min[q(X1), q(X2)]	Nonlinear Weakening
min[q(X1), q(X2)] < q(X1∩X2) < max[q(X1), q(X2)]	Uni-linear Weakening
q(X1∩X2) = q(X1) + q(X2)	Independent
max[q(X1), q(X2)] < q(X1∩X2) < q(X1) + q(X2)	double-factor Enhancement
q(X1∩X2) > q(X1) + q(X2)	Nonlinear Enhancement

**Table 4:** Confusion matrix for the accuracy test of the mangrove species classification results.

Classification Results	Field Data						
	*A. marina*	*R. stylosa*	*A. corniculatum*	*B. gymnorrhiza*	*K. obovata*	Total	Producer′s Accuracy (%)
Unclassified	771	82	40	0	12	905	85.19
*A. marina*	70,668	0	0	0	0	70,668	100
*R. stylosa*	0	6711	0	22	0	6733	99.67
*A. corniculatum*	7	54	2693	190	5	2949	91.32
*B. gymnorrhiza*	0	109	221	2880	0	3210	89.72
*K. obovata*	0	0	31	0	1137	1168	97.35
Total	71,446	6956	2985	3092	1154	85,633	-
User′s accuracy (%)	98.91	96.48	90.22	93.14	98.53	-	-
Overall accuracy (%)	98.18						
Kappa	0.94						

**Table 5:** Estimation models for AGB across different mangrove species.

Species	Model Equation (kg/Pixel)	R^2^	Adjusted R^2^	D-W	Sig.
*A. marina*	107.49 − 163.87EVI + 587.52NDVI − 257.67SAVI	0.780	0.733	2.258	0.000
*B. gymnorrhiza*	394.56 + 106.10P_COR2_	0.405	0.346	1.645	0.026
*R. stylosa*	541.58 + 216.16P_HO2_	0.780	0.548	1.638	0.034
*A. corniculatum*	7.64 + 3.23P_COR1_ + 2.11P_HO2_ + 8.32SAVI	0.434	0.340	1.704	0.007
*K. obovata*	49.33 + 12.06P_SE1_ + 28.40P_VA1_ + 34.27P_VA2_	0.777	0.693	2.131	0.006

**Table 6:** Estimation models for BGB across different mangrove species.

Species	Model Equation (kg/Pixel)
*A. marina*	0.52 × 101.00 log_10_ (107.49 − 163.87EVI + 587.52NDVI − 257.67SAVI)
*B. gymnorrhiza*	1.15 × 100.79 log_10_ (394.56 + 106.10$P_{\mathrm{COR}2}$)
*R. stylosa*	1.06 × 100.86 log_10_ (541.58 + 216.16$P_{\mathrm{HO}2}$)
*A. corniculatum*	3.83 × 100.69 log_10_ (7.64 + 3.23$P_{\mathrm{COR}1}$+ 2.11$P_{\mathrm{HO}2}$ + 8.32SAVI)
*K. obovata*	0.16 × 101.39 log_10_ (49.33 + 12.06$P_{\mathrm{SE}1}$+ 28.40$P_{\mathrm{VA}1}$+ 34.27$P_{\mathrm{VA}2}$)

**Table 7:** Overall model fit indices test.

Name	Evaluation Criteria	Test Results	
χ^2^/DF	<3 Satisfactory; <5 Acceptable	8	Together with other indicators
GFI	>0.9 Acceptable	0.977	Acceptable
AGFI	>0.9 Acceptable	0.940	Acceptable
PGFI	>0.5 Acceptable	0.372	Together with other indicators
TLI	>0.9 Acceptable; >0.95 Satisfactory	0.927	Acceptable
NFI	>0.9 Acceptable; >0.95 Excellent	0.961	Excellent
CFI	>0.9 Acceptable; >0.95 Satisfactory	0.961	Excellent
RMSEA	<0.05 Excellent; 0.05–0.08 Satisfactory; 0.08–0.10 Edge; >0.10 Poor-fitting	0.065	Satisfactory

## Data Availability

Dataset available on request from the authors.

## References

[1] Zhang J., Gan S., Yang P., Zhou J., Huang X., Chen H., He H., Saintilan N., Sanders C.J., Wang F. (2024). A Global Assessment of Mangrove Soil Organic Carbon Sources and Implications for Blue Carbon Credit. Nat. Commun..

[2] Friedlingstein P., Le Quéré C., O’sullivan M., Hauck J., Landschützer P., Luijkx I.T., Li H., van der Woude A., Schwingshackl C., Pongratz J. (2026). Emerging Climate Impact on Carbon Sinks in a Consolidated Carbon Budget. Nature.

[3] Allen C.D., Macalady A.K., Chenchouni H., Bachelet D., Mcdowell N., Vennetier M., Kitzberger T., Rigling A., Breshears D.D., Hogg E.H.T. (2010). A Global Overview of Drought and Heat-Induced Tree Mortality Reveals Emerging Climate Change Risks for Forests. For. Ecol. Manag..

[4] Wu L., Guo E., An Y., Xiong Q., Shi X., Zhang X., Sun Z. (2023). Evaluating the Losses and Recovery of GPP in the Subtropical Mangrove Forest Directly Attacked by Tropical Cyclone: Case Study in Hainan Island. Remote Sens..

[5] International Science Council (2025). 10 New Insights in Climate Science. https://council.science/news/10-new-insights-in-climate-science/.

[6] Ali S., Dey G., Hoang N., Nuong K., Rahman A., Wang L., Sukul U., Das K., Kumar R., Wang S. (2025). Earth-Science Reviews Carbon Sequestration in Mangrove Ecosystems: Sources, Transportation Pathways, Influencing Factors, and Its Role in the Carbon Budget. Earth-Sci. Rev..

[7] Piñeiro-Juncal N., Mateo M.Á., Leiva-dueñas C., Serrano E., Inostroza K., Soler M., Apostolaki E.T., Lavery P., Duarte C.M., Lafratta A. (2025). Soil Organic Carbon Depth profiles and Centennial and Millennial Decay Rates in Tidal Marsh, Mangrove and Seagrass Blue Carbon Ecosystems. Commun. Earth Environ..

[8] Selvaraj J.J., Pérez B.E.G. (2023). Heliyon Estimating Mangrove Aboveground Biomass in the Colombian Pacific Coast: A Multisensor and Machine Learning Approach. Heliyon.

[9] Prakash A.J., Behera M.D., Ghosh S.M., Das A., Mishra D.R. (2022). A New Synergistic Approach for Sentinel-1 and PALSAR-2 in a Machine Learning Framework to Predict Aboveground Biomass of a Dense Mangrove Forest. Ecol. Inform..

[10] Li L., Liu W., Ai J., Cai S., Dong J. (2023). Predicting Mangrove Distributions in the Beibu Gulf, Guangxi, China, Using the MaxEnt Model: Determining Tree Species Selection. Forests.

[11] Wang K., Jia M., Zhang X., Zhao C., Zhang R., Wang Z. (2024). Evaluating Ecosystem Service Value Changes in Mangrove Forests in Guangxi, China, from 2016 to 2020. Remote Sens..

[12] Document Resource (2025). HYT Technical Specification for Survey and Assessment of Blue Carbon Ecosystem Carbon Stocks—Mangroves. https://www.doc88.com/p-77180460426086.html.

[13] Yao Y., Liang S., Fisher J.B., Zhang Y., Cheng J., Chen J., Jia K., Zhang X., Bei X., Shang K. (2021). A Novel NIR—Red Spectral Domain Evapotranspiration Model from the Chinese GF-1 Satellite: Application to the Huailai Agricultural Region of China. IEEE Trans. Geosci. Remote Sens..

[14] Patra S., Mishra M. (2025). Spatio-Temporal Changes in Mangroves of Bhitarkanika National Park along the Eastern Coast of India over a Three Decades Period Using Remote Sensing Spectral Index NDVI. Geol. Ecol. Landsc..

[15] Huete A.R. (1988). A Soil-Adjusted Vegetation Index (SAVI). Remote Sens. Environ..

[16] Huete A., Didan K., Miura T., Rodriguez E.P., Gao X., Ferreira L.G. (2002). Overview of the Radiometric and Biophysical Performance of the MODIS Vegetation Indices. Remote Sens. Environ..

[17] Papi F. (2025). Turning Seasonal Signals into Segmentation Cues: Recolouring the Harmonic Normalized Difference Vegetation Index for Agricultural Field Delineation. Sensors.

[18] Gitelson A.A., Kaufman Y.J., Merzlyak M.N. (1994). Use of a Green Channel in Remote Sensing of Global Vegetation from EOS-MODIS. Remote Sens. Environ..

[19] Lu Y., Yang C. (2024). Influence of GLCM Texture Parameters on Lithological Mapping Using Sentinel-1 Imagery. Geocarto Int..

[20] Ali U.A.E., Maniamfu P., Kameyama K. (2024). Efficient Band Reduction for Hyperspectral Imaging with Dependency-Based Segmented Principal Component Analysis. Int. J. Remote Sens..

[21] Computer Network Information Center, Chinese Academy of Sciences Geospatial Data Cloud. https://www.gscloud.cn/.

[22] Arnaud M., Lovelock C.E., Maceiras M., Thuong-Huyen D., Robin S., Abiven S., Mishra A.K., Farooq S.H., Bhadra T., Felbacq A. (2025). The Nature of Soil Blue Carbon Varies across Mangrove Geomorphic Settings. Commun. Earth Environ..

[23] Biswas J., Biswas N. (2026). Improved Remote Sensing Ecological Index for Monitoring Urban Sustainability: 100 Resilient Cities vs Non-Resilient Cities in South Asia. Environ. Monit. Assess..

[24] Rovai A.S., Twilley R.R., Castañeda-Moya E., Midway S.R., Friess D.A., Trettin C.C., Bukoski J.J., Stovall A.E., Pagliosa P.R., Fonseca A.L. (2021). Macroecological Patterns of Forest Structure and Allometric Scaling in Mangrove Forests. Glob. Ecol. Biogeogr..

[25] Ponce-Bobadilla A.V., Schmitt V., Maier C.S., Mensing S., Stodtmann S. (2024). Practical Guide to SHAP Analysis: Explaining Supervised Machine Learning Model Predictions in Drug Development. Clin. Transl. Sci..

[26] Jiang M., Qiu P., Wang D., Wu M., Lai R., Zou X., Si T., Li H., Shi Q., Lin Y. (2025). Influence of Micro-Topography on the Spatial Heterogeneity of above-Ground Biomass: Lessons from the Qinglan Port Mangrove Nature Reserve, China. Ecol. Indic..

[27] Gu Y., Zhang Y., Akihara M., Zhang Y., Huang H. (2025). The Spatial and Temporal Characteristics and Influencing Factors of Intangible Cultural Heritage in Fujian Province. Land.

[28] He Q.F., Zheng W., Huang X.R., Liu X., Shen W.H., He F. (2017). Carbon Storage and Distribution Characteristics of Mangroves in Qinzhou Bay, Guangxi. J. Cent. South Univ. For. Technol..

[29] Kauffman J.B., Donato D.C. (2012). Protocols for the Measurement, Monitoring and Reporting of Structure, Biomass and Carbon Stocks in Mangrove Forests.

[30] Zheng W.J., Lin P., Xue X.Z., Lu C.Y., Zheng F.Z., Yin Y. (1995). Dynamics of C, H and N in Rhizophora stylosa Mangrove Forest in Guangxi. Chin. J. Appl. Ecol..

[31] Kang L., Huang H.M., Ran Y., Peng B. (2025). Carbon Storage Potential and Influencing Factors of Mangrove Plantation in Kaozhouyang, Guangdong Province, South China. Front. Mar. Sci..

[32] Mahmood H., Siddique M.R.H., Abdullah S.M.R., Costello L., Matieu H., Iqbal Z., Akhter M. (2019). Which Option Best Estimates the Above-Ground Biomass of Mangroves of Bangladesh: Pantropical or Site- and Species-Specific Models ?. Wetl. Ecol. Manag..

[33] Analuddin K., Helmi M., Pribadi R., Adrianto L., Jaya L.G., Iba W., Susetyo Adi N., Septiana A., Nadaoka K., Nakamura T. (2024). Mangrove Vulnerability and Blue Carbon Storage in the Coral Triangle Areas, Southeast Sulawesi, Indonesia. Front. Ecol. Evol..

[34] Li G., Zhuo T., You X. (2025). Assessment of global planted-to-natural mangroves biomass ratio and mangroves biomass carbon stocks by machine learning. Environ. Res..

[35] Tian Y., Huang H., Zhou G., Zhang Q., Tao J., Zhang Y., Lin J. (2021). Aboveground Mangrove Biomass Estimation in Beibu Gulf Using Machine Learning and UAV Remote Sensing. Sci. Total Environ..

[36] Kamal M., Hidayatullah M.F., Mahyatar P., Ridha S.M. (2022). Estimation of Aboveground Mangrove Carbon Stocks from WorldView-2 Imagery Based on Generic and Species-Specific Allometric Equations. Remote Sens. Appl. Soc. Environ..

[37] Li H.Y., Jia M.M., Zhang R., Ren Y.X., Wen X. (2019). Incorporating the Plant Phenological Trajectory into Mangrove Species Mapping with Dense Time Series Sentinel-2 Imagery and the Google Earth Engine Platform. Remote Sens..

[38] Wang X.Z., Tang L.L., Fu J.C. (2023). Performance Evaluation of Mangrove Species Classification Based on Multi-Source Remote Sensing Data Using Extremely Randomized Trees in Fucheng Town, Leizhou City, Guangdong Province. Remote Sens..

[39] Chen Y., Shen X., Yan C., Jiang B.Q., Li R.L., Chai M.W. (2026). Triple-Feature Fusion from UAV Multispectral Imagery Enhances Species-Level Mangrove Carbon Assessment. Sci. Rep..

[40] Zhang X.H., Yin Z.Q., Chen Y.P., Li F.Y., Fu C.W., Wang Y.F. (2025). Carbon Stock Spatial Patterns in Mangroves of Shankou, Guangxi. IEEE J. Sel. Top. Appl. Earth Obs. Remote Sens..

[41] Feng B., Tao Y., Xie X., Qin Y., Hu B., Jia R., Pan L., Liu W., Jiang W. (2024). Identification of Suitable Mangrove Distribution Areas and Estimation of Carbon Stocks for Mangrove Protection and Restoration Action Plan in China. J. Mar. Sci. Eng..

[42] Dong D., Huang H., Gao Q., Li K., Zhang S., Yan R. (2025). Integrating Remote Sensing and Field Data to Quantify Mangrove Biomass Carbon Hotspots and Protection Gaps in Guangdong, China. Forests.

[43] Bai J., Meng Y., Gou R., Lyu J., Dai Z., Diao X., Zhang H., Luo Y., Zhu X., Lin G. (2021). Mangrove Diversity Enhances Plant Biomass Production and Carbon Storage in Hainan Island, China. Funct. Ecol..

[44] Kang Y., Assavapanuvat P., Kaplan D.A., Osland M.J. (2026). Variation in Soil Organic Carbon Across a Latitudinal Chronosequence of Mangrove Poleward Expansion. Ecosystems.

[45] Rovai A.S., Twilley R.R., Castañeda-moya E., Riul P., Cifuentes-jara M., Manrow-villalobos M., Horta P.A., Simonassi J.C., Fonseca A.L., Pagliosa P.R. (2018). Global controls on carbon storage in mangrove soils. Nat. Clim. Change.

[46] Alongi D.M. (2014). Carbon Sequestration in Mangrove Forests. Carbon Manag..

[47] Huang X., Chen Y., Mo W., Fan H., Liu W., Sun M., Xie M., Xu S. (2021). Variation of Climate and Its Influencing Factors in Mangrove Ecological Zones of Beibu Gulf, Guangxi over the Past 60 Years. Acta Ecol. Sin..

[48] Simard M., Fatoyinbo L., Smetanka C., Rivera-monroy V.H., Castañeda-moya E., Thomas N., Stocken T.V. (2019). Mangrove canopy height globally related to precipitation, temperature and cyclone frequency. Nat. Geosci..

